# MaMADS2 repression in banana fruits modifies hormone synthesis and signalling pathways prior to climacteric stage

**DOI:** 10.1186/s12870-018-1480-5

**Published:** 2018-11-06

**Authors:** Esther Yakir, Fei Zhangjun, Noa Sela, Yimin Xu, Vikram Singh, Anurag Dagar, Janak Raj Joshi, Maren Müller, Sergi Munné-Bosch, James J. Giovannoni, Julia Vrebalov, Haya Friedman

**Affiliations:** 1 Department of Postharvest Science of Fresh Produce, Agricultural Research Organization (ARO), The Volcani Center, P.O. Box 6, 50250 Bet Dagan, Israel; 2 Boyce Thompson Institute for Plant Research and USDA-ARS Robert W. Holley Center, Tower Road, Cornell Campus, Ithaca, NY USA; 3 Plant Pathology and Weed Research, ARO, The Volcani Center, Bet Dagan, Israel; 4 Departament de Biologia Vegetal, Facultat de Biologia, Universitat de Barcelona, Avinguda Diagonal, 645, E-08028 Barcelona, Spain

**Keywords:** Abscisic acid, Auxin, Gibberellic acid, Hormones, Jasmonic acid, Salicylic acid

## Abstract

**Background:**

While the role of ethylene in fruit ripening has been widely studied, the contributions of additional plant hormones are less clear. Here we examined the interactions between the transcription factor MaMADS2-box which plays a major role in banana fruit ripening and hormonal regulation. Specifically, we used MaMADS2 repressed lines in transcriptome and hormonal analyses throughout ripening and assessed hormone and gene expression perturbations as compared to wild-type (WT) control fruit.

**Results:**

Our analyses revealed major differences in hormones levels and in expression of hormone synthesis and signaling genes mediated by MaMADS2 especially in preclimacteric pulp. Genes encoding ethylene biosynthesis enzymes had lower expression in the pulp of the repressed lines, consistent with reduced ethylene production. Generally, the expression of other hormone (auxin, gibberellins, abscisic acid, jasmonic acid and salicylic acid) response pathway genes were down regulated in the WT pulp prior to ripening, but remained high in MaMADS2 repressed lines. Hormone levels of abscisic acid were also higher, however, active gibberellin levels were lower and auxin levels were similar with MaMADS2 repression as compared to WT. Although abscisic level was higher in MaMADS2 repression, exogenous abscisic acid shortened the time to ethylene production and increased MaMADS2 mRNA accumulation in WT. Exogenous ethylene did not influence abscisic acid level. CRE - a cytokinin receptor, increased its expression during maturation in WT and was lower especially at prebreaker in the repressed line and zeatin level was lower at mature green of the repressed line in comparison to WT.

**Conclusions:**

In addition to previously reported effects of MaMADS2 on ethylene, this transcription factor also influences other plant hormones, particularly at the pre-climacteric stage. The cytokinin pathway may play a previously unanticipated role via MaMADS2 in banana ripening. Finally, abscisic acid enhances *MaMADS2* expression to promote ripening, but the transcription factor in turn auto inhibits ABA synthesis and signaling. Together, these results demonstrate a complex interaction of plant hormones and banana fruit ripening mediated by MaMADS2.

**Electronic supplementary material:**

The online version of this article (10.1186/s12870-018-1480-5) contains supplementary material, which is available to authorized users.

## Background

Fruits of plants in the genus *Musa* including bananas and plantains represent staple foods for millions of people, especially in developing countries, and an important carbohydrate and nutrient source for billions more, the world over. Although multiple genetic components of ripening control have been discovered, especially in the model plant tomato [1–3], the mechanism in banana is less characterized, though critical transcription factors have recently been discovered [4, 5]. Banana, as typical of climacteric fruit, exhibit an increase in respiration and a surge in ethylene production concomitant with ripening [6, 7].

The involvement of ethylene in ripening has been established, but less so the role of other hormones: indole-3-acetic acid (IAA or auxin), cytokinin (CK), abscisic acid (ABA), gibberellins (GAs), jasmonic acid (JA) and salicylic acid (SA) [8–10]. Auxin is involved in early fruit development, but its role in ripening is less clear. Contradicting conclusions as a negative or positive regulator were reported based on its exogenous application [11], including in banana [12] and from its measurements or gene-related expression [13, 14]. By analyzing the function of ARF genes it was concluded that auxin may play a complex role in fruit ripening possibly affecting other hormones [15, 16]. Moreover, it has been shown in tomato that auxin may specifically intersect with the ethylene response pathway at the point of EIN2 [17]. So, while the role of auxin remains ambiguous and may vary based on development, tissue or species, available evidence, nevertheless, indicates involvement in fruit ripening.

Gibberellins (GAs) have been mostly associated with fruit growth [18], with little evidence regarding a specific role in fruit ripening. Interestingly, exogenous GA_3_ did not alter the respiration or the ethylene profile of banana, but delayed starch breakdown and sucrose accumulation, possibly by reducing levels of sucrose phosphate synthase [19].

ABA most likely play a positive role in ripening of climacteric and non-climacteric fruit [20]. Abscisic acid (ABA) was found to increase prior to the increase of ethylene in tomato [21]. ABA levels and the expression of *9-cis-epoxycarotenoid dioxygenase* (*NCED*), encoding a critical enzyme in ABA biosynthesis, increased during fruit maturation, and declined during ripening as ethylene increased [22]. External application of ABA to tomato and banana promoted ripening [22, 23], and this effect is consistent with ethylene induction [8, 24]. Silencing tomato *SlNCED* decreased fruit softening and increased shelf life, but increased ethylene production [25], supporting a complex role in both ethylene synthesis and response.

Several studies suggest that jasmonates (JAs) might be positive regulators of fruit ripening through induced expression of ethylene synthesis pathway genes [18, 26, 27]. Salicylic acid (SA) levels have not been determined during ripening, however application of exogenous SA to different fruits including banana, reduced respiration and ethylene production and decreased cell wall deterioration [28, 29]. Finally, cytokinins (CKs) are usually associated with delayed senescence, cell death [30] and fruit ripening [31], and their levels have been reported to decrease with tomato development [18, 32]. However, tomato plants overexpressing the *isopentenyl transferase* (*ipt*) gene, encoding a key enzyme in the CKs biosynthesis, presented a sectored ripening phenotype [33] and in grapes, the levels of CK increased following veraison and remained high during ripening [34], Hence, the role of CKs in ripening is still obscure. While some evidence exists for multiple hormones having effects on ripening, the molecular mechanism(s) through which they coordinate and balance their effects and their interactions with the ripening-related transcription factors remain uncertain.

We have previously shown that transgenic *MaMADS2* repressed fruits are delayed in ripening [4] and thus, may present a system for addressing hormone roles in the ripening process. Here we characterize in wild type (WT) and *MaMADS2* repressed lines the transcriptomes in peel and pulp through fruit ripening and hormones levels in pulp. Our analysis revealed that hormone synthesis and signaling pathways, and hormone levels have been altered in response to MaMADS2 repression prior to normal ethylene induction, suggesting a role for MaMADS2 in coordinating hormone cross-talk at preclimacteric stage.

## Methods

### Plant material and sample collections

Banana (Musa acuminate, AAA Cavendish subgroup, cv. Grand Nain) WT and *MaMADS2* repressed lines (antisense line #44 and RNAi line #32) were grown in the Jordan Valley, and fruits were collected for transcriptome, hormone analysis or ABA and ethylene treatment. Fruit were harvested at 75% of final cross-sectional filling and fruit maturity, determined by measuring the average of the locular angles of banana fruit and the ratio of peel to pulp in cross sections [35]. Hands of the first, second or third tiers, containing 10–30 fruit were separated from the bunch and stored at 20 °C and 95% RH. Samples of both control and transformed banana were collected every 2–3 days and color, ethylene (C_2_H_4_) production and carbon dioxide (CO_2_) emission were determent. Fruits of different ripening stages: mature green (MG; 1 day after harvest), prebreaker (PB; 5(WT), 9(#44) and 10(#32) days after harvest) just before the ethylene peak, breaker (B; 7(WT), 12(#44) and 17(#32) days after harvest) and ripe (R; 10(WT), 17(#44) and 20 (#32) days after harvest) with brown spots were analyzed in RNAseq. The stages were determined by the color of the peel using Minolta. The average of hue (o) from at least two replicates for WT, *MaMADS2* antisense (#44) and *MaMADS2* RNAi (#32), were: MG:118, 119.3, 119.3; PB:106.3, 108, 113.3; B:100.5, 96.1, 99.1; R: 86.2, 88.4, 89, respectively.

### ABA and ethylene treatments

Slices of WT banana fingers taken from MG stage were treated with ABA. Slices were placed on a Whatman paper immersed with 10 μM ABA or 0.01% methanol in a petri dish. Ethylene production rate was followed periodically by transferring the slices to a 120 ml sealed jar, and mRNA was extracted from three days-treated and non-treated slices. For ethylene treatment, slices of WT banana fingers were taken from MG and PB and placed in a 120 ml sealed glass jars at 20 °C with 1 ppm ethylene for 24 h. ABA was extracted by acidic methanol extraction as described [36].

### Ripening measurements

Ethylene and carbon dioxide production were determined by GC head-space gas analysis of individual banana fingers in 2-L sealed glass jars at 20 °C as described [5]. Peel color was determined from surface area of at least two individual banana fingers using a Minolta CR-300 colorimeter (Minolta Corporation, New Jersey, USA). Total soluble solids (TSS) of peel and pulp juice, resulting from freezing and thawing of the tissues, was determined using a handheld HSR-500 refractometer (Atago Co. Ltd., Japan).

### Transcriptome analysis

The transcriptomes of WT, as well as, *MaMADS2* repressed lines were determined by RNAseq analysis. Total RNA was extracted using the Spectrum™ Plant Total RNA Kit (Sigma Aldrich) and preparation of samples for RNAseq library construction is described along with procedures for sequencing and sequence data analysis with levels of expression determined as Reads per Kilo base of transcript per Million mapped reads (RPKM) [37]. As an independent secondary confirmation of RNAseq data, several genes were also analyzed by Quantitative reverse transcriptase PCR (qRT-PCR).

### Comparative transcriptome analysis and differential gene expression

Gene expression was normalized and differential expression was calculated using the R Bioconductor [38] “DESeq” package [39]. The results are displayed in BaseMean (mean normalized counts, averaged over all replicates). The transcript reference of banana was downloaded from the banana genome hub website (http://banana-genome.cirad.fr/) [40] and were further annotated by blasting the transcripts against the NCBI non-redundant protein database [41] with the aid of blast2go software [42]. The translation of chromosome location of each of the genes to genes number is summarized in Additional file 1: Table S1. The correlation among all the conditions (peel/pulp, stages of ripening: MG/PB/B/R, genotypes: WT/#44/#32) is calculated based on the normalized count table with all the average expression of all the genes and not only the differentially expressed. The correlation takes into consideration the array of expression matrix of every gene in each condition and the distance matrix is calculated. The correlation between gene expressions in different developmental stages was calculated with “dist” function in R applying the “Canberra” method. The distance matrix was visualized using gplots R package. Functional analysis of the differentially modified genes (FDR < 0.1) was performed by Ontologizer (Parent-Child-Union, Benjamini-Hochberg) to detect enrichment of GO (Gene Ontology) http://ontologizer.de/ [43].

### Hormones profiling

The hormonal profile of WT, as well as *MaMADS2* repressed line (#32) fruit tissues, was determined by UHPLC-MS/MS as described [44]. Samples were collected at several developmental stages: MG (1 day after harvest), PB (5(WT) and 14(#32) days after harvest) and B (10(WT) and 20 (#32) days after harvest). The pulp of six banana fingers was analyzed for each stage. The stages were determined by the color of the peel using Minolta (see above). The average of hue (o) for WT and *MaMADS2* repressed (#32), were: MG:120.3, 120.9; PB:108.9, 111; B:98.6, 99.2, respectively.

### Hormonometer analysis

The Hormonometer was developed as a tool in *Arabidopsis thaliana* to identify the transcriptome footprint of several hormones and their temporal induction [45]. We have identified 10,606 genes with homology of more than 60% identity to *Arabidopsis thaliana* genes. This “Arabidopsis homologs gene pool” was used for the Hormonometer analysis. The fold change in expression between transgenic lines and WT, as well as, the *p*-value was loaded to Hormonometer tool (https://hormonometer.weizmann.ac.il/hormb/about) and the output gave the correlation values between the query gene pool and the transcriptome footprint for each hormone [45]. High positive values for a specific hormone indicates that there is high representation of the specific hormone-induced genes within the query gene pool.

## Results

### Peel and pulp transcriptome diverged during ripening and *MaMADS2* repression delayed maturation

To better assess the role of MaMADS2 during ripening, we performed RNA-seq transcriptome analysis on WT and *MaMADS2* repressed fruit at the same physiological stages based on peel color. This approach enabled assessment of whether the underlying transcriptomes were the same or different at each stage which would in turn, indicates whether most or a subset of ripening phenomena were influenced by MaMADS2. The transcriptomes of WT, and the *MaMADS2* repressed lines; *MaMADS2* RNAi (#32) and antisense (#44) lines were assessed throughout post-harvest ripening at the mature-green (MG; average H°: 118, 119.3, 119.3 respectively), pre-breaker (PB; average H°: 106.3, 113.3, 108 respectively), breaker (B; average H°: 100.5, 99.1, 96.1 respectively) and ripe (R; average H°: 86.2, 89, 88.4 respectively) stages.

**Fig. 1:**
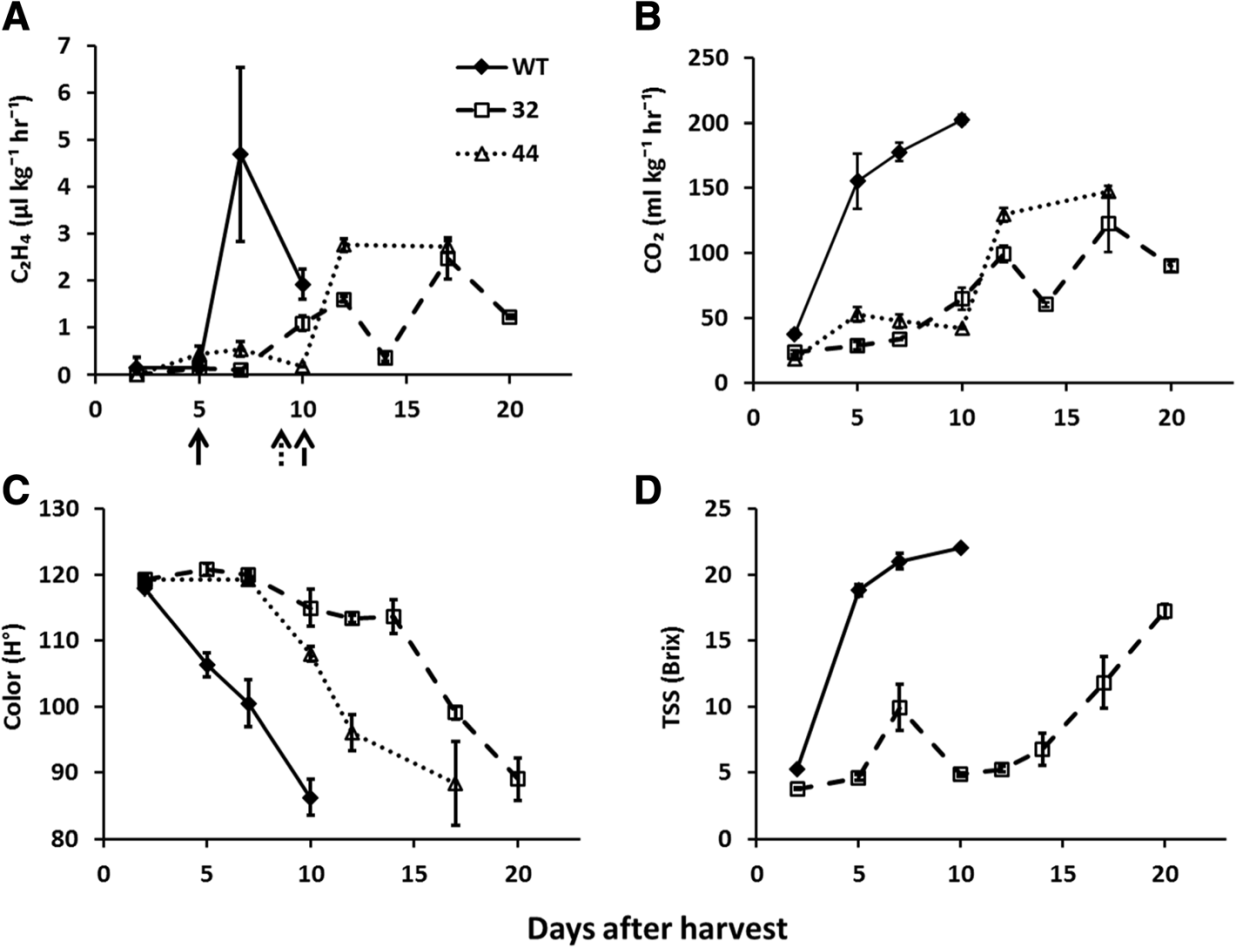
Ethylene production, respiration rate, color and total soluble solids (TSS) in WT and *MaMADS2* repressed banana fruits after harvest. Ethylene production rate (**a**), CO_2_ production rate (**b**), color expressed as hue angle (o) (**c**) and total soluble solids expressed as brix (%) (**d**) were measured in fruits of the second hand of WT, *MaMADS2* RNAi line (#32) and *MaMADS2* antisense line (#44) during ripening. For each time point at least three fruits were measured and average and standard error plotted. Arrow in panel A indicate the samples taken as PB in each of the lines

Bananas of lines #32 and #44 (Fig. 1) were late in all ripening parameters examined: CO_2_, ethylene production, color and TSS. The ethylene peak in WT fruits occurred 7 days after harvest, and in lines #32 and #44 it was delayed by 10 and 5 days, respectively (Fig. 1A). CO_2_ levels increased 5 days after harvest in WT fruits, but it was delayed by 7 days in the transgenic fruits (Fig. 1B). As with ethylene levels, WT fruits became yellow 7 days after harvest (100.5 ± 3.5H°), while the yellow color was delayed by 10 and 5 days in lines #32 and #44, respectively (Fig. 1C).

**Fig. 2:**
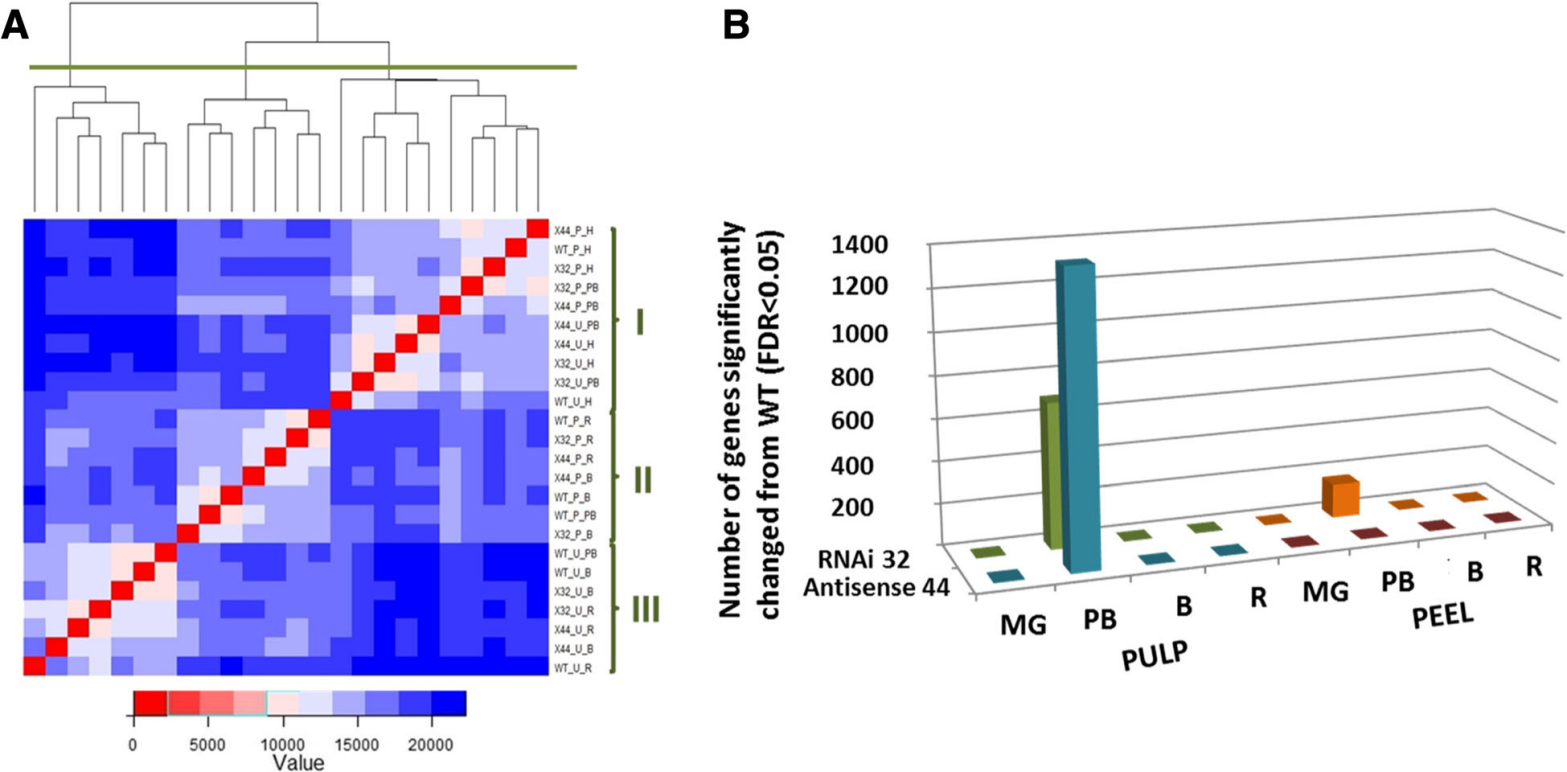
Transcriptome differences between WT and *MaMADS2* repressed banana fruits during ripening. **a**. Heatmap of the normalized gene expression averages of all samples and hierarchical clustering of all samples. On right the different samples clustered into three groups (I, II and III). The analysis was performed on samples taken from peel (P) and pulp (U) of WT (WT) and repressed lines *MaMADS2* antisense (#44) and *MaMADS2* RNAi (#32) at harvest/mature green (H), prebreaker (PB), breaker (B) and ripe (R). the color scale shows the distances between different conditions; red show low distance while blue larger distance; **b**. Number of genes significantly changed (FDR < 0.05) between #32 or #44 lines and WT fruits in peel and pulp in the developmental stages of MG, PB, B and R

Fig. 2A presents a heat map and a hierarchical clustering of gene expression across tissues, developmental stages and genotypes. This analysis revealed major differences in PB between WT and the *MaMADS2* repressed lines, but almost no differences at MG or breaker/ripe, supporting that the fruits from the repressed lines and WT were in a similar physiological stages at MG and B/R in both peel and pulp (Fig. 2B). Three different expression clusters are defined as I, II and III (Fig. 2A). Cluster I includes all the expression averages of samples taken immediately after harvest (MG) from WT and the two transgenic plants both peel and pulp. Cluster I also contains expression averages of samples taken at PB from the pulp and peel of lines #32 and #44, but not from PB of WT. Hence, this cluster represents the least physiologically mature tissues. Cluster II includes all the expression averages of samples taken from peel at B and at R and the expression averages of samples taken from WT peel at PB. Effectively, it contains all the ripening peel samples. Cluster III includes all the expression averages of samples taken from pulp at B and at R and the expression averages of samples taken from WT pulp at PB and hence it contains all the ripening pulp samples. Hence, the transcriptome profiles of WT during PB of peel and pulp are closer to the transcriptome profiles of these tissues at B, than to that of MG. In contrast, the transcriptome profiles during PB of line #32 and line #44 pulp and peel are closer to the transcriptome profiles of MG, than to that of B.

### Transcriptome analysis revealed elevated expression of hormone-related genes at the pre-breaker stage in *MaMADS2* repressed lines

Comparison of expression data of WT and *MaMADS2* repressed lines #32 and #44 revealed a significant difference in gene expression levels for the PB stage in pulp and to a lesser degree in the peel, and no significant change between the WT and the transgenic plants in MG, B and R (Fig. 2B). This further supports the fact that all lines were collected at a comparable developmental stage and that transgenic fruits achieved full ripeness, although later.

In the peel of line 32 the expression of 162 genes was significantly changed at PB in comparison to WT (FDR < 0.05), and more than 90% of these genes showed the same direction of change in the peel of line #44 (increase or decrease), with larger FDR. This likely reflects the less severe phenotype of line #44 as compared to line #32. In the pulp of lines #32 and #44 the expression of 692 and 1374 genes, respectively, were significantly changed at PB (Additional file 1: Fig. S1A). Interestingly, although line #32 showed a more delayed phenotype than line #44, fewer genes were significantly changed in its pulp. Perhaps the parameters of fruit ripening reflecting mainly the events which occur in the peel (color change, ethylene production), which were the base for transcriptome analysis sampling, do not fully capture pulp ripening outcomes. Most of the genes that were changed in the pulp of #32 were also significantly changed in the pulp of line #44 and the rest exhibited the same directional change. Similarly, up to 99% of the genes that were altered in the pulp of line #44 showed the same directional change in #32 line. Based on this consistency, the restriction imposed on selection of the genes from line #32 was reduced (FDR < 0.1) and 1216 modified genes from this line were used in subsequent analysis (Additional file 1: Fig. S1B). Hence, the transcriptome analysis was performed on 1685 genes which constitute the union between the modified genes in #32 and #44 pulp at PB.

Out of 1685 modified genes, 545 and 1140 genes showed lower and higher expression, respectively, in both lines #44 and #32 compared to WT at PB. Gene Ontologizer (GO) was applied to identify functional groups (Additional file 1: Fig. S2A, B). Among the genes exhibiting lower expression in *MaMADS2* repressed lines, there were groups related to protein assembly and to cytoskeleton and organelle movement, indicating that these processes are induced by MaMADS2 and likely associated with ripening (Additional file 1: Fig. S2A). Among the genes exhibiting higher expression, were groups related to RNA metabolism and gene expression, transport, oxidative stress, various biosynthetic pathways and hormonal signaling (Additional file 1: Fig. S2B), indicating that these processes are negatively influenced by MaMADS2. Further analysis of the hormonal signaling pathways was performed by KEGG on the genes included in the 1685 differentially expressed gene pool. This analysis identified putative proteins involved in the hormonal synthesis and response pathways of ethylene, ABA, IAA, CK, GA, SA and JA.

### MaMADS2 influences ethylene synthesis and response pathways genes

**Fig. 3:**
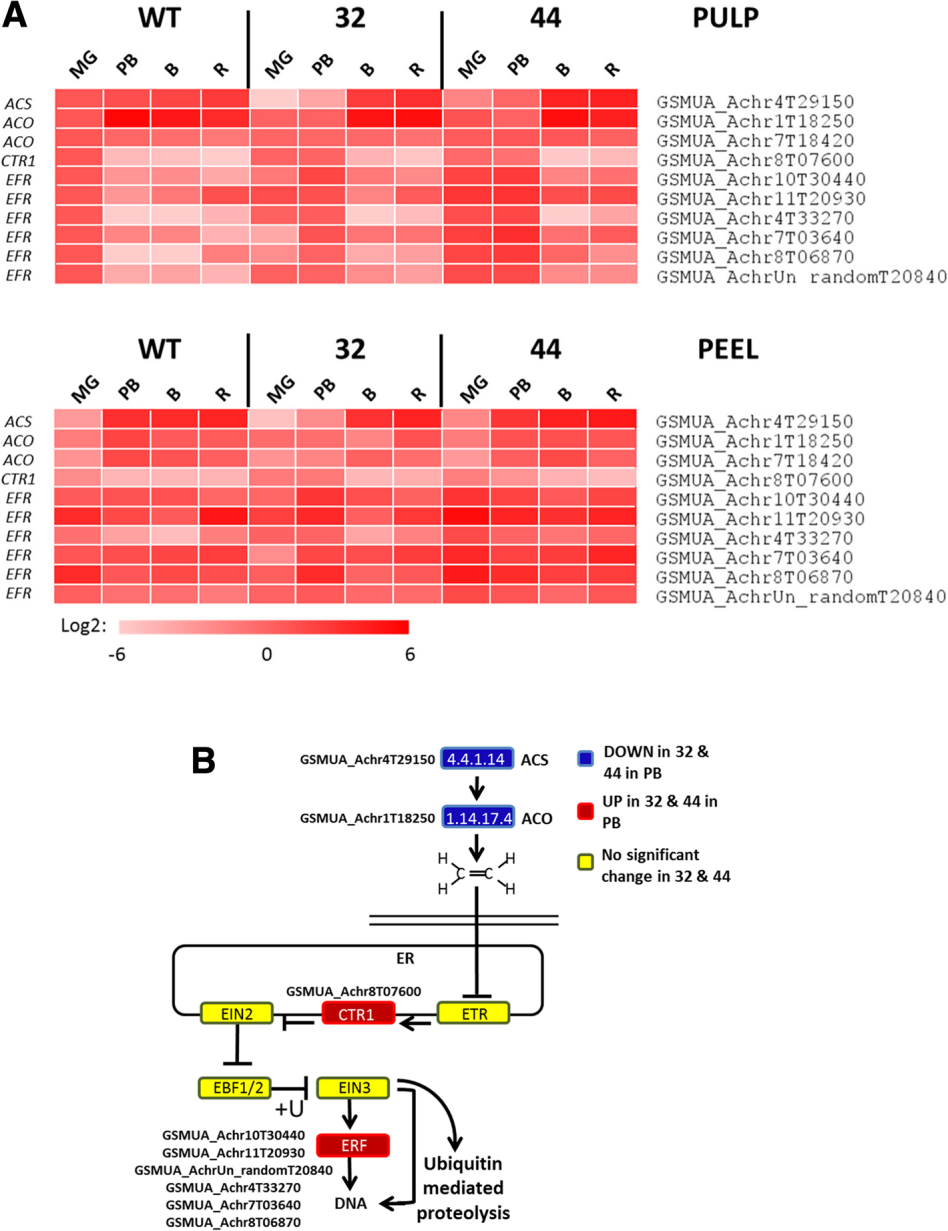
Ethylene biosynthesis and signaling pathway in WT and *MaMADS2* repressed fruits. **a**. Heat map of the genes changed in PB between WT and the *MaMADS2* repressed lines. Each gene is normalized to the expression level in WT at MG; **b**. The ethylene biosynthesis and signaling pathway scheme. The genes’ expression significantly higher (red) or lower (blue) in #32 or #44 fruits in comparison to WT at PB. Yellow color indicates no change. Detailed description of genes is provided at [47]

Genes of the ethylene biosynthesis pathway have significantly lower expression in *MaMADS2* repressed lines than in WT at PB in pulp (Fig. 3). One homolog of *SlACS2* (*GSMUA_Achr4T29150*) involved in tomato ripening ethylene biosynthesis [46], is expressed in the pulp of WT at MG and its expression increased by three fold at PB (Fig. 3A), and a similar expression pattern was observed in the WT peel (Fig. 3A). In contrast, the level of *GSMUA_Achr4T29150* in the pulp of *MaMADS2* repressed lines, was significantly lower at MG and PB (less than 4% and about 30%, for lines #32 and #44, respectively), and also in the peel of line #32 at PB stage (Fig. 3A, B, Additional file 2: Table S2).

Fifteen *ACO* homologs were expressed in banana fruits with expression levels above 50 BaseMean. Two tomato *SlACO1* homologs, which contribute to ethylene production during ripening [46], were identified in banana fruits and one, *GSMUA_Achr1T18250*, is also significantly lower at PB in *MaMADS2* repressed lines in comparison to WT (Fig. 3A, B, Additional file 2: Table S2). Another *SlACO1* homolog, *GSMUA_Achr7T18420* (*ACO1*) is highly expressed (100 times more than the others *ACO* genes) and it is higher in WT than in the repressed lines only in the peel (Additional file 2: Table S2). Other *ACO* genes presented a diversity of expression patterns in both peel and pulp, however, most of the induced genes showed earlier induction in WT than in the repressed lines (Additional file 1: Fig. S3).

KEGG analysis also identified a banana homolog of the negative regulator of the tomato ethylene response pathway *SlCTR1* [47]. *CTR1* homologue *GSMUA_Achr8T07600* is expressed in WT at MG in both pulp and peel but decreases sharply to 3% and 18% at PB, respectively (Fig. 3A, Additional file 2: Table S2). This gene is highly expressed in the pulp and the peel of the *MaMADS2* repressed lines at MG, but also at PB and decrease at B (Additional file 2: Table S2). Downstream in the ethylene response pathway, a group of *ERF* (*Ethylene Response Element*) homologs were also highly expressed in the pulp of *MaMADS2* repressed lines at PB. One of them, *GSMUA_Achr8T06870* (*ERF11*), was previously demonstrate to represses the expression of *ACO1* [48].

### MaMADS2 influences ABA synthesis and response

**Fig. 4:**
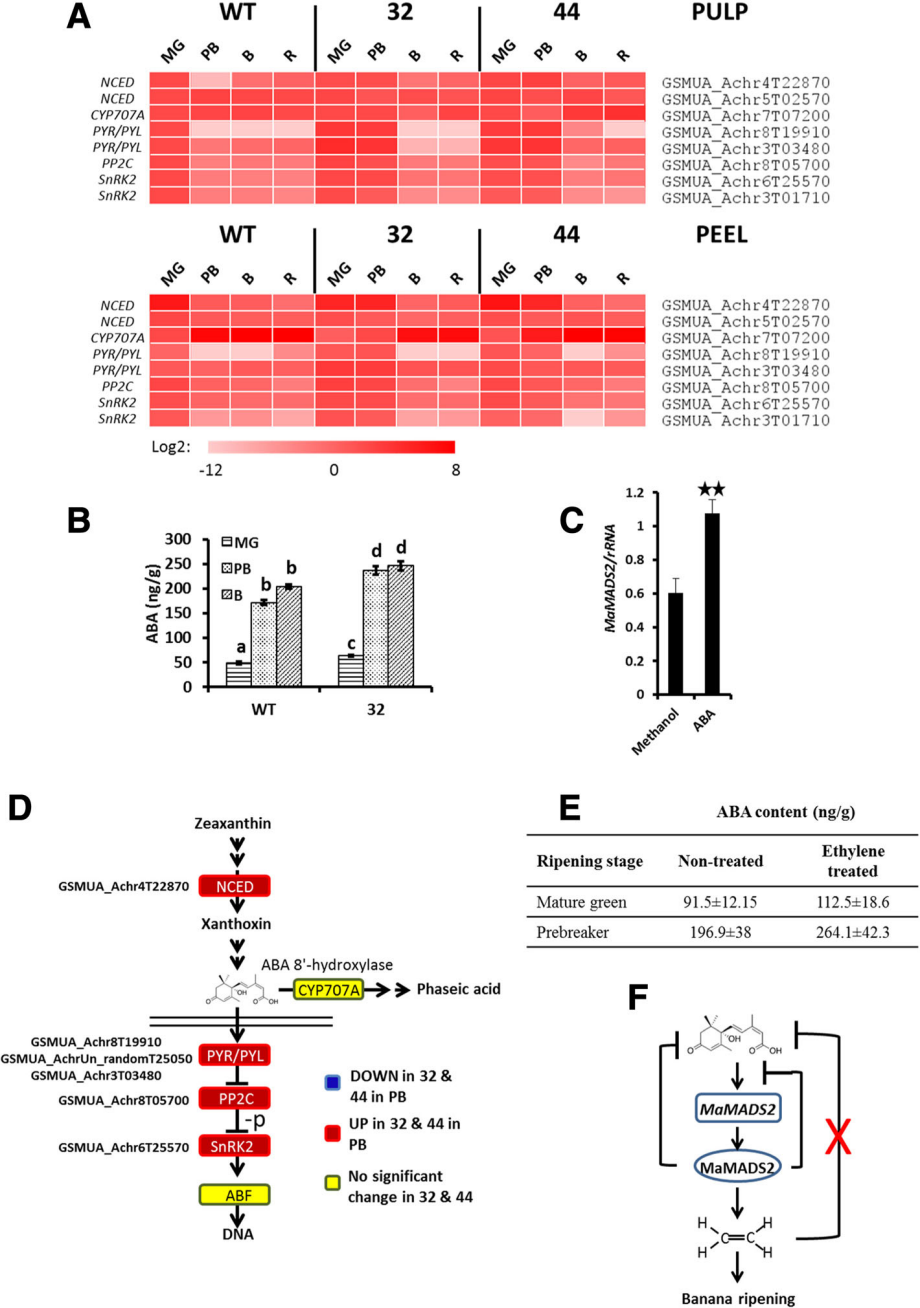
ABA biosynthesis and signaling pathway and ABA level in WT and *MaMADS2* repressed fruits. **a**. Heat map of the genes changed in PB between WT and the *MaMADS2* repressed lines. Each gene is normalized to the expression level in WT at MG; **b**. ABA level in WT and #32 pulps; **c**. The average of *MaMADS2* expression levels in WT after ABA treatment (**: *p* < 0.01 standard error is presented); **d**. The ABA biosynthesis and signaling pathway scheme. The genes’ expression significantly higher (red) or lower (blue) in #32 or #44 fruits in comparison to WT at PB. Yellow color indicates no change. Detailed description of genes is provided by [50, 51]; **e**. Effect of ethylene treatment on ABA content in banana slices; **f**. A suggested model for the interactions of ABA with *MaMADS2* and ethylene: rectangular: transcript, oval: protein, X: interaction that was rejected based on our results

ABA level is dependent on the rate of biosynthesis and catabolism. The *9-cis-epoxycarotenoid Dioxygenases* (*NCED*) encodes an enzyme involved in ABA biosynthesis, while *CYP707A*, encodes a hydroxylase that converts ABA to 8′-OH-ABA and hence is involved in ABA degradation [49, 50]. In the transcriptome data there are two expressed *NCED* homologs and the expression of one, *GSMUA_Achr4T22870*, was low in pulp of WT and significantly higher in *MaMADS2* repressed lines at PB (Fig. 4A, D, Additional file 2: Table S2). The expression of another *NCED* gene *GSMUA_Achr5T02570* increased during ripening in WT, but oscillated in the *MaMADS2* repressed lines with high levels at MG. In addition, one *CYP707A* was found to be highly expressed in the banana peel (*GSMUA_Achr7T07200*), but it had low expression in the repressed lines at PB, as well as, in the pulp of WT (Fig. 4A, Additional file 2: Table S2).

In agreement with *GSMUA_Achr4T22870* and *GSMUA_Achr5T02570* expression, the ABA levels increase in pulp and peel (Fig. 4B and unpublished data) at the transition from MG (average H°: WT 120.3 and line #32 120.9) to PB (average H°: WT 108.9 and line ##32 111) in all lines prior to ethylene peak which occurred at B (Fig. 1) and were higher in the *MaMADS2* repressed lines than in WT in pulp at all stages (Fig. 4B).

To elucidate the interactions between ethylene, ABA and *MaMADS2-box*, WT banana slices were treated with ABA and the effect on ethylene and *MaMADS2* expression was examined. Ethylene production peak occurred on the fifth day following ABA treatment, and the level was 5.42 ± 0.75 μl/kg*h, while the peak in non-ABA treated slices occurred on the seventh day and the level was 3.75 ± 0.22 μl/kg*h. ABA also increased the expression of *MaMADS2* (Fig. 4C). The reciprocal experiment showed, however, that ethylene treatment did not increase ABA levels (Fig. 4E).

Homologs of the ABA response pathway: ABA receptor, *PYRABACTIN RESISTANCE1* (*PYR/PYL*) (*GSMUA_Achr8T19910*, *GSMUA_AchrUn_randomT25050*, and *GSMUA_Achr3T03480*); Type 2C protein phosphatases (*PP2C*) which activates the SnRK2 and is inactivated by binding to the receptor (*GSMUA_Achr8T05700*) and the sucrose non fermenting (SNF1)-related protein kinase (*SnRK2*) which phosphorylate proteins involved in ABA response (*GSMUA_Achr6T25570* and *GSMUA_Achr3T01710*) [50, 51] were expressed in the pulp at WT MG but declined thereafter (Fig. 4A, Additional file 2: Table S2). A similar pattern was observed in the peel (Fig. 4A). The expression of these genes was significantly higher in both pulp and peel at PB in the repressed lines than in WT and was reduced later on (Fig. 4A).

In order to examine if there is an ABA-related transcriptome footprint in WT and the transgenic lines, the “Arabidopsis homologs gene pool” (see Methods) was analyzed by the Hormonometer [45]. The analysis showed high correlations between the query gene pool and ABA transcriptomic footprint of *Arabidopsis thaliana* (Fig. 9). This indicates that both transgenic lines have higher expression of ABA-induced genes at BP compared to WT.

### MaMADS2 regulates Zeatin level and response

**Fig. 5:**
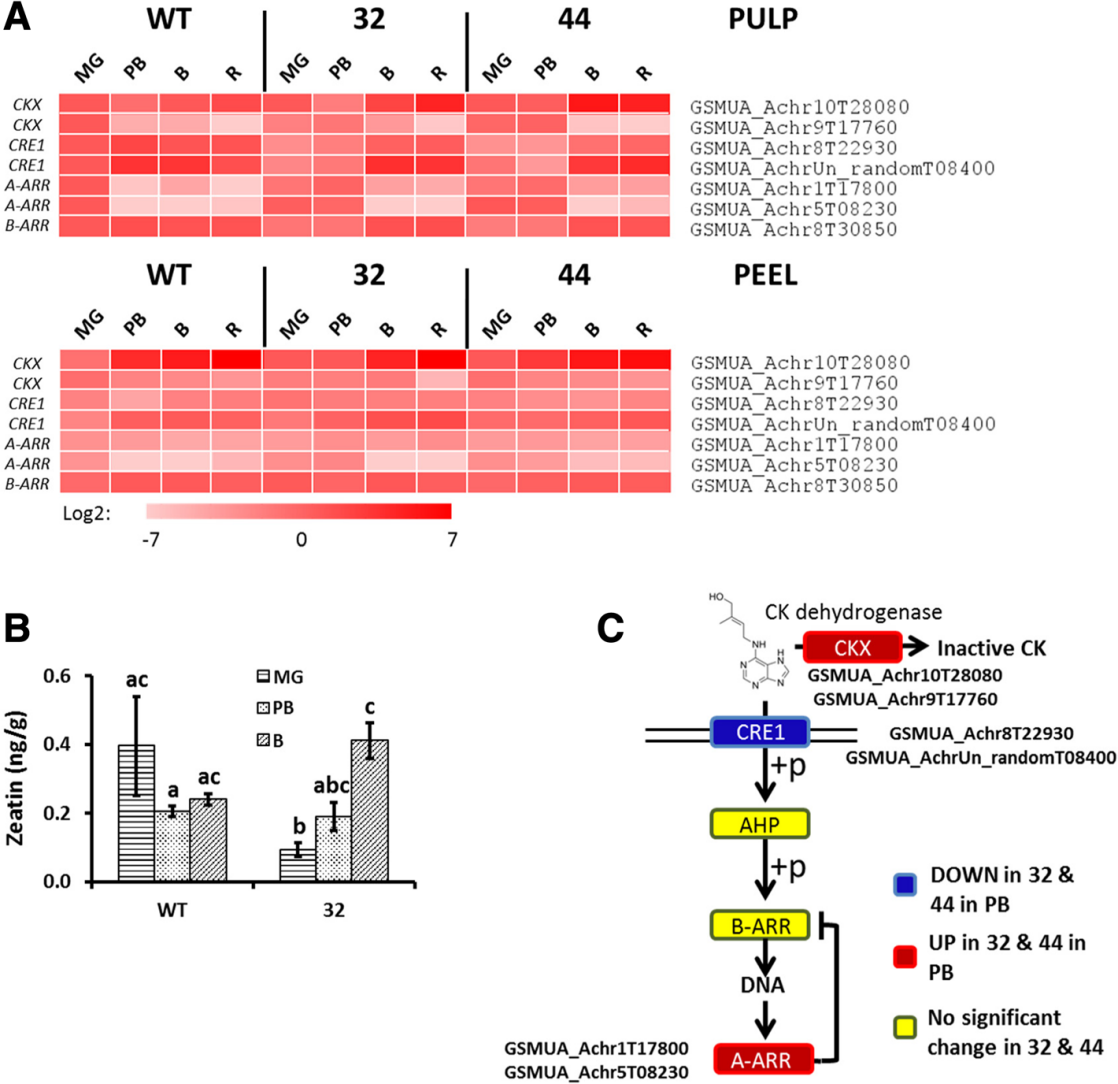
CK signaling pathway and zeatin level in WT and *MaMADS2* repressed fruits. **a**. Heat map of the genes changed in PB between WT and the *MaMADS2* repressed lines. Each gene is normalized to the expression level in WT at MG; **b**. Zeatin level in WT and #32 pulps; **c**. The CK signaling pathway scheme. The genes’ expression significantly higher (red) or lower (blue) in #32 or #44 fruits in comparison to WT at PB. Yellow color indicates no change. Detailed description of genes is provided at [55]

CK levels are determined by the biosynthesis and degradation enzymes Isopentenyl transferases and CK dehydrogenase (CKX), respectively [52]. Two homologs of CK dehydrogenase (CKX), *GSMUA_Achr10T28080* and *GSMUA_Achr9T17760*, were significantly modified through ripening and in transgenic lines (Fig. 5A). *GSMUA_Achr10T28080* was hardly expressed in WT in the pulp, but was expressed in the repressed lines. *GSMUA_Achr9T17760*, was expressed at MG in WT pulp and peel, and in pulp it decreased at PB, but in the repressed lines the decline was delayed (Fig. 5A, Additional file 2: Table S2).

Zeatin was the only detectable CK in our analysis (Fig. 5B). In WT there was no significant change during ripening, however, the levels of zeatin were lower in *MaMADS2* suppressed line than in WT, and gradually increased during ripening in this line (Fig. 5B).

Plants respond to CK by using a phospho-relay mechanism which involves the membrane two component receptor, Cytokinin Response (CRE1) [53] and downstream transcription activators A/B-Arabidopsis Response Regulators (ARR) [54, 55]. Consistent with lower zeatin levels in *MaMADS2* repressed lines than in WT, two *CRE1* homologs (*GSMUA_Achr8T22930* and *GSMUA_AchrUn_randomT08400*) clearly exhibited lower expression in the repressed lines than in WT pulp at PB (Fig. 5A, C and Additional file 2: Table S2). In addition, the expression levels of all other 7 *CRE1* homologs expressed in ripening fruits was lower in the pulp of the repressed lines than in WT (more than twice the levels) at MG and/or PB (Additional file 1: Fig. S4).

On the other hand, the expression of *A-ARR* homologs (*GSMUA_Achr1T17800* and *GSMUA_Achr5T08230*) was higher at PB in the pulp of the repressed lines in comparison to WT (Fig. 5A, C and Additional file 2: Table S2). It has been shown that the A-ARR are induced by CK and they repress their own expression [56], shutting down the CK response [57]. Moreover, the only *ARR-B* homologe, *GSMUA*_*Achr8T30850*, that was highly expressed in banana fruits was lower (less than 20%) in the pulp of repressed lines compared with WT at PB (Fig. 5A and Additional file 2: Table S2). Hormonometer analysis [45] showed high negative correlation between the query gene pool and a CK response footprint, indicating that the cytokinin-related transcriptomic footprint is lower in the transgenic lines compared to WT.

### MaMADS2 has a dual effect on auxin response pathway(s)

**Fig. 6:**
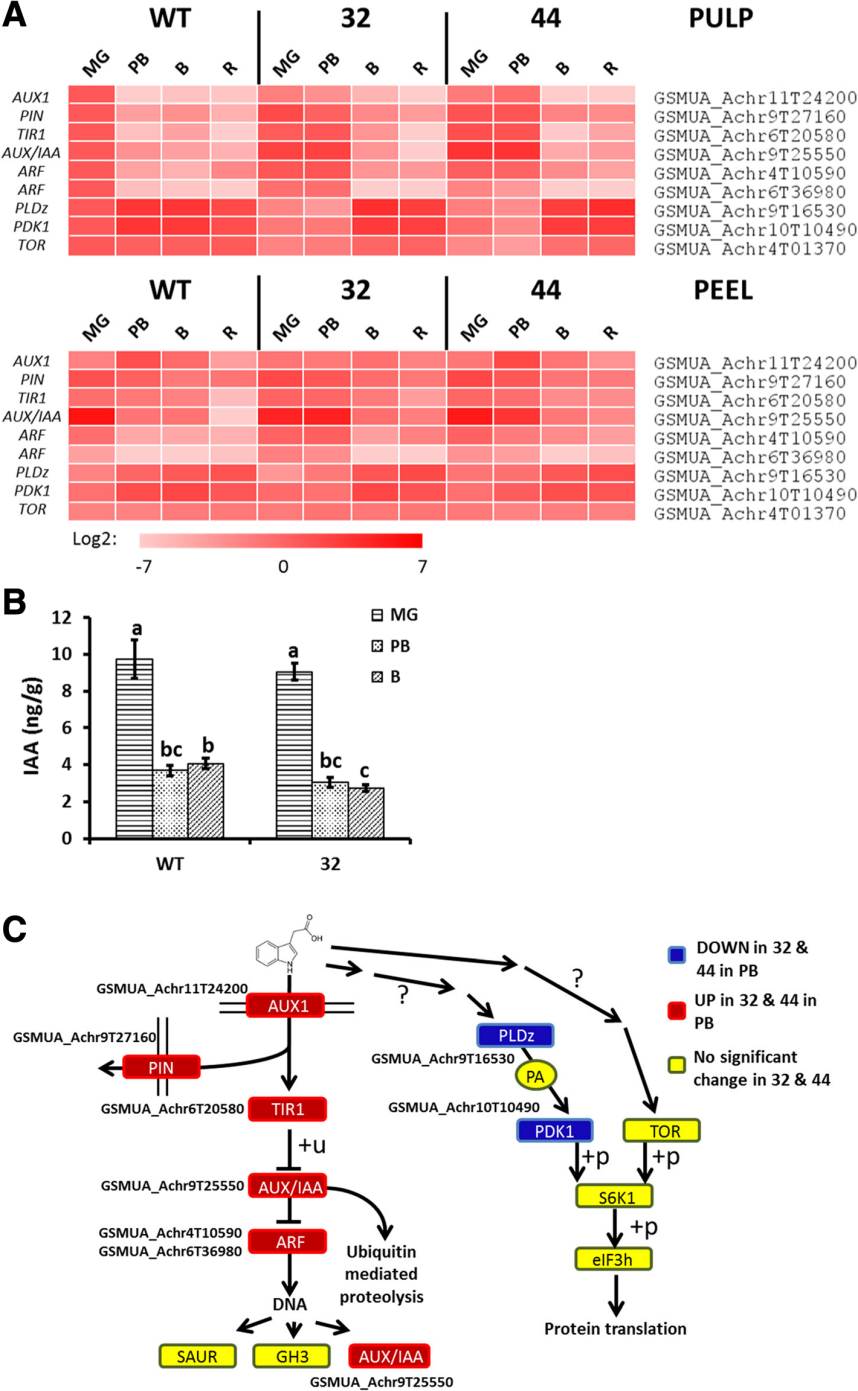
IAA signaling pathway and IAA level in WT and *MaMADS2* repressed fruits. **a**. Heat map of the genes changed in PB between WT and the *MaMADS2* repressed lines. Each gene is normalized to the expression level in WT at MG; **b**. IAA level in WT and #32 pulps; **c**. The IAA signaling pathway scheme. The genes’ expression significantly higher (red) or lower (blue) in #32 or #44 fruits in comparison to WT at PB. Yellow color indicates no change. Detailed description of genes is provided at [58, 59]

There are no differences in IAA levels between WT and *MaMADS2* repressed lines, and the levels of auxin was high at MG, declined at PB and remained low during maturation (Fig. 6B). Key elements of auxin transport and signaling pathways were modified in the repressed lines (Fig. 6A, C). Auxin transport is controlled by influx (AUX) and efflux carriers (PIN) and gene transcription is regulated through the TIR-AUX/IAA-ARF pathway (Fig. 6C) [58]. The transcript expression of auxin transport components and TIR-AUX/IAA-ARF signaling pathway *GSMUA_Achr11T24200* (*AUX1*), *GSMUA_Achr9T27160* (*PIN1*), *GSMUA_Achr6T20580* (*TIR1*), *GSMUA_Achr9T25550* (*AUX/IAA*) and *GSMUA_Achr4T10590* and *GSMUA_Achr6T36980* (*ARF*) genes were significantly higher in the pulp of repressed lines at PB in comparison to WT (Fig. 6A, C and Additional file 2: Table S2). This is primarily due to a delay in the reduction of these transcripts during ripening in the transgenic lines. All of these genes exhibited high expression levels at MG in WT pulp, and most of them also in peel, and their expression declined at PB to a very low levels, yet the decline was delayed to B in the repressed lines (Fig. 6A and Additional file 2: Table S2). In addition, all 10 homologs of *TIR1* in banana follow this profile in both peel and pulp (Additional file 1: Fig. S5).

Recently, another poorly understood pathway involving protein translation has been suggested to contribute to auxin response [59] (Fig. 6C). This pathway is comprised of TARGET OF RAPAMYCIN (TOR) and Phospholipase D zeta (PLDz) which produce a lipid-related second messenger, phosphatidic acid (PA), that activates the PHOSPHOLIPID-DEPENDENT PROTEIN KINASE 1 (PDK1) involved in RIBOSOMAL S6 KINASE (S6K) regulation of protein translation [59]. Transcript homologs of several genes in this pathway, *GSMUA_Achr9T16530* (*PLDz*), *GSMUA_Achr10T10490* (*PDK1*) and *GSMUA_Achr4T01370* (*TOR*), showed elevated expression during ripening and were much lower in the pulp of *MaMADS2* repressed lines at PB (Fig. 6A, C and Additional file 2: Table S2). In the peel there were no differences in the expression of these genes between WT and the repressed lines.

### MaMADS2 modifies GA synthesis and response

**Fig. 7:**
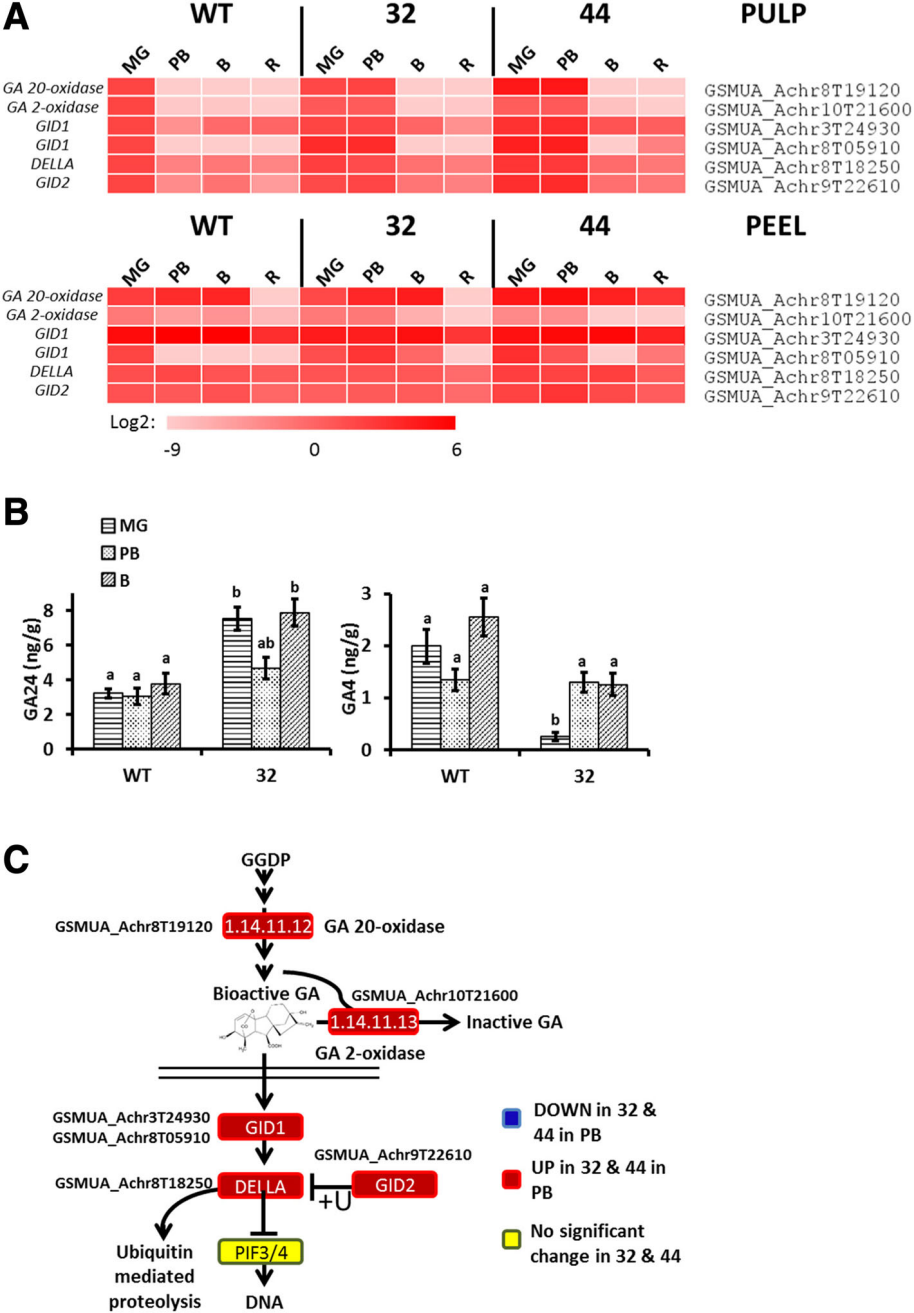
GA biosynthesis and signaling pathway and GA levels in WT and *MaMADS2* repressed fruits. **a**. Heat map of the genes changed in PB between WT and the *MaMADS2* repressed lines. Each gene is normalized to the expression level in WT at MG; **b**. GA24 and GA4 levels in WT and #32 pulps; **c**. The GA biosynthesis and signaling pathway scheme. The genes’ expression significantly higher (red) or lower (blue) in #32 or 44 fruits in comparison to WT at PB. Yellow color indicates no change. Detailed description of genes is provided at [60, 61]

The compounds GA_19_, GA_24_, GA_9_, and GA_20_ are precursors of the active GA_4_/GA_1_ and GA_3_/GA_7_ [60]. Analysis of GA levels in the pulp of WT and transgenic plant revealed that, while GA_9_ level was similar in both WT and the repressed line in the pulp (Additional file 1: Fig. S6B), that of GA_24_ was higher and that of GA_4_ was lower at MG in the repressed line in comparison to WT (Fig. 7B). GA_1_, GA_19_, and GA_20_ could not be detected. These results coincide with the expression levels of genes encoding enzymes of biosynthesis and degradation of GA. GA 20-oxidase is responsible for the synthesis of the GA precursors [60] and the expression of its homolog *GSMUA_Achr8T19120* was very low in WT pulp at all stages, and slightly higher in the peel reaching a peak at B stage (Fig. 7A and Additional file 2: Table S2). However, in the repressed line in both pulp and peel the expression was higher at MG and PB, possibly explaining the higher GA_24_ precursor in the repressed line. The expression of *GSMUA_Achr10T21600*, a homolog of GA 2-oxidase, which deactivates GA [60] decreased sharply after MG in the WT pulp. However, in both repressed lines the expression in pulp remained high through PB (Fig. 7A and Additional file 2: Table S2). This likely explains the low levels of GA_4_ observed in pulp of *MaMADS2* suppress line.

GA signaling includes *GIBBERELLIN-INSENSITIVE DWARF1* (*GID1/2*) genes encoding gibberellins receptors which mediate the degradation of the negative regulator DELLA [61]. The expression of *GSMUA_Achr3T24930* and *GSMUA_Achr8T05910* (*GID1*), *GSMUA_Achr9T22610* (*GID2*) and *GSMUA_Achr8T18250* (*DELLA*) was higher in the *MaMADS2* repressed lines pulp at MG and PB in comparison to WT (Fig. 7A, C and Additional file 2: Table S2). Moreover, out of six additional *DELLA* homologs three were expressed and their levels were higher in the pulp of repressed line at MG and PB than in WT (Additional file 1: Fig. S6A).

Combining high levels of *DELLA* homologs functioning as negative regulator(s) [61], with the low levels GA_4_ in the *MaMADS2* suppressed lines, indicates that GA response is likely reduced in the transgenic lines. However, a Hormonometer analysis [45] of the query gene pool showed non-conclusive results; while there was negative correlation between banana query gene pool with 3 h GA_3_-induced response, there was a positive correlation with 6 and 9 h GA_3_-induced response (Fig. 9).

### MaMADS2 influences JA and SA levels as well as their response pathways

**Fig. 8:**
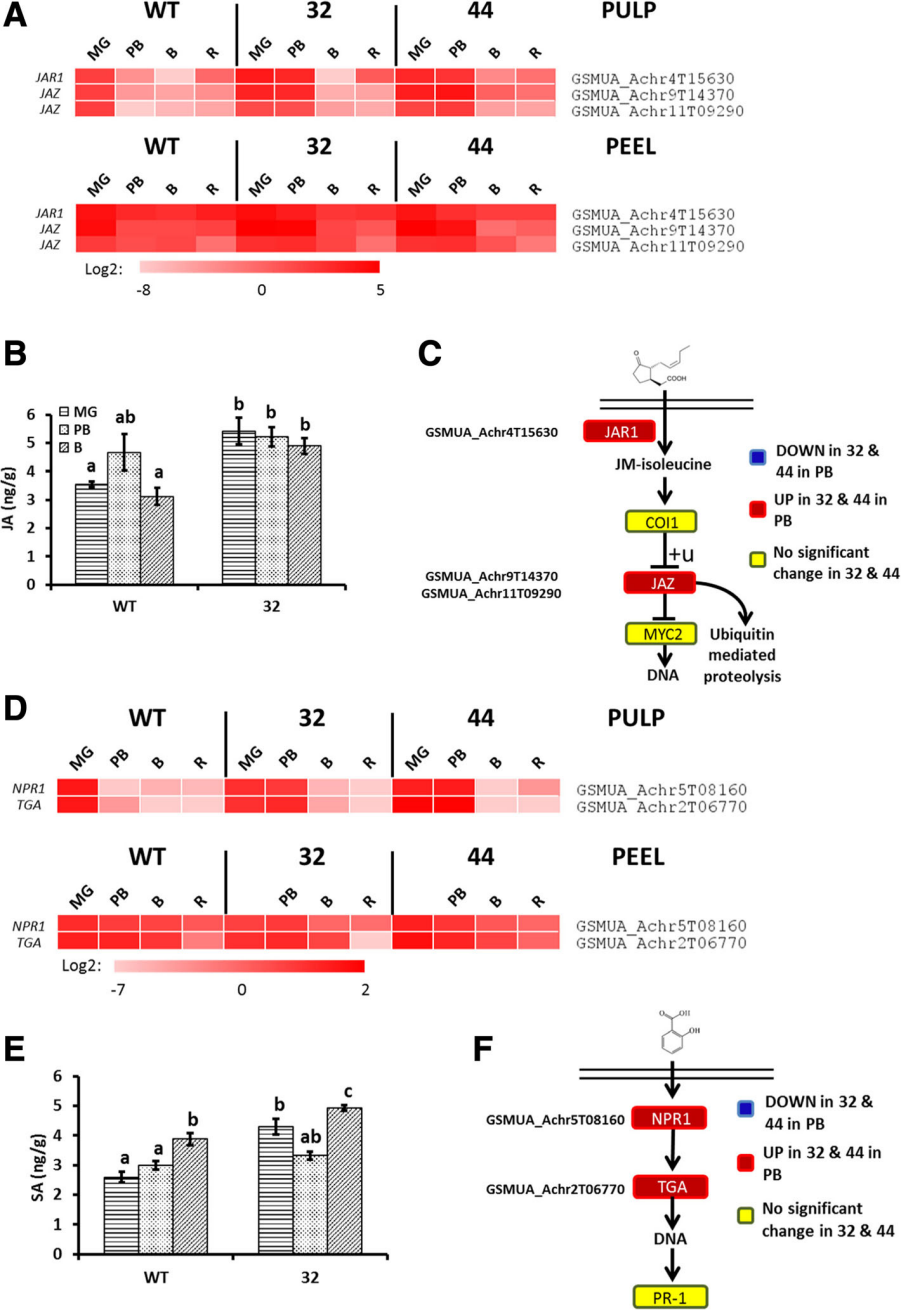
JA and SA signaling pathway and JA and SA levels in WT and *MaMADS2* repressed fruits. **a**. Heat map of the genes in JA signaling pathway changed in PB between WT and the *MaMADS2* repressed lines. Each gene is normalized to the expression level in WT at MG; **b**. JA level in WT and #32 pulps; **c**. The JA signaling pathway scheme. The genes’ expression significantly higher (red) or lower (blue) in #32 or #44 fruits in comparison to WT at PB. Yellow color indicates no change; **d**. Heat map of the genes in SA signaling pathway changed in PB between WT and the *MaMADS2* repressed lines. Each gene is normalized to the expression level in WT at MG; **e**. SA level in WT and #32 pulps; **f**. The SA signaling pathway scheme. The genes’ expression significantly higher (red) or lower (blue) in #32 or #44 fruits in comparison to WT at PB. Yellow color indicates no change. Detailed description of genes is provided at [62] for JA and at [63] for SA

**Fig. 9:**
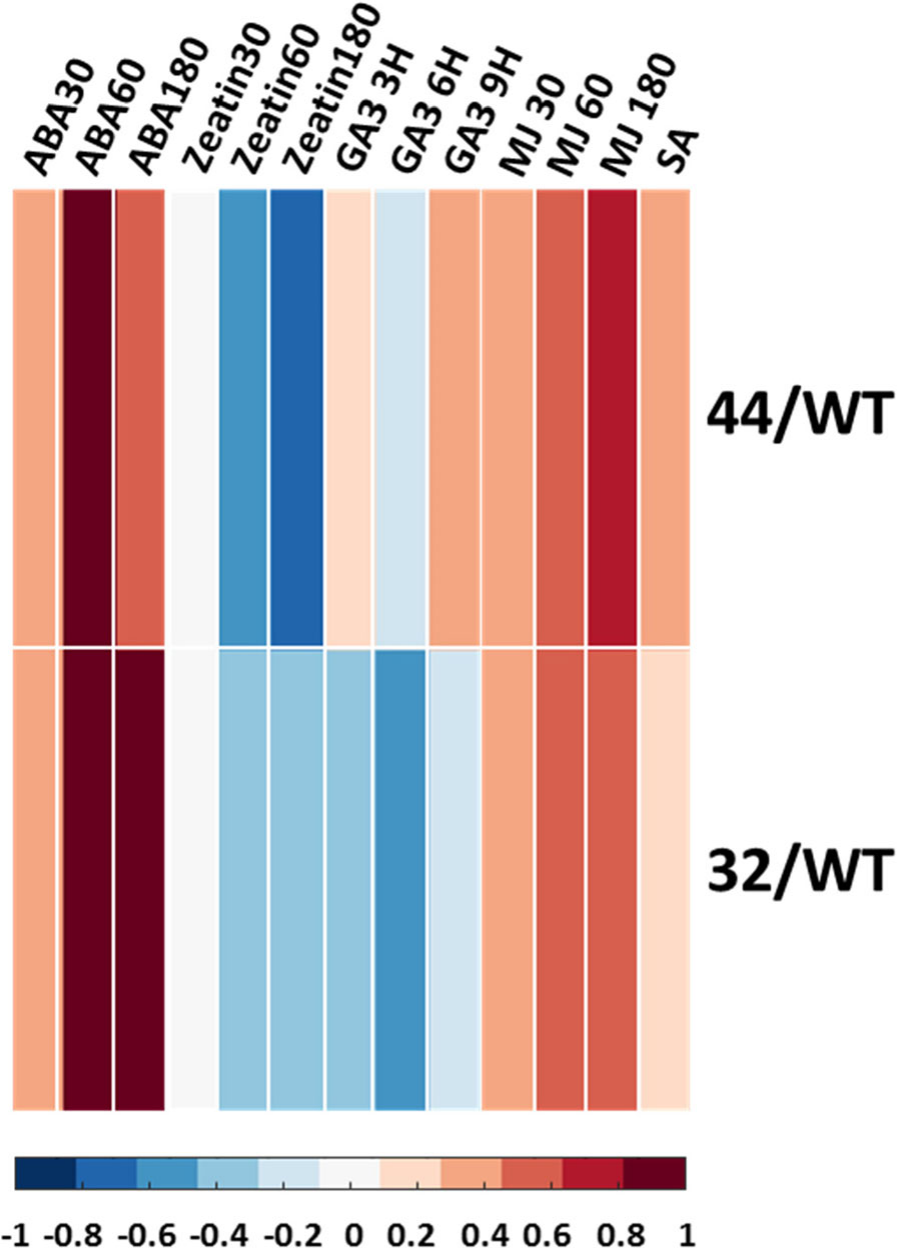
Difference in hormonal footprint between *MaMADS2* repressed lines and WT fruits. Hormonometer analysis of the “Arabidopsis homologs gene pool” (see methods) expressed in *MaMADS2* repressed lines in comparison to WT. Dark brown indicates strong hormone footprint in *MaMADS2* repressed line and blue indicates strong hormone footprint in WT.

The levels of JA and SA were significantly higher in *MaMADS2* repressed lines than in WT in most ripening stages (Fig. 8B, E). The transcripts of the JA biosynthesis and JA and SA responses pathways, exhibited higher expression in the repressed lines in the pulp at PB (Fig. 8A, C, D, F and Additional file 2: Table S2). The expressions in WT pulp of *GSMUA_Achr4T15630* (*JAR1* homolog, involved in formation of an active JA conjugate) [62] and *GSMUA_Achr9T14370* and *GSMUA_Achr11T09290* (*JAZ* homologs, negative regulator of the pathway) [62] are mostly low in WT pulp throughout ripening, but elevated in the pulp of the repressed lines (Fig. 8A, C and Additional file 2: Table S2). The Hormonometer analysis showed a positive correlation between the query gene pool and the JA- transcriptome foot print, indicating a relatively high JA-related transcriptome foot print of the transgenic lines (Fig. 9).

The SA signaling pathway includes two major genes *NON-EXPRESSOR OF PATHOGENESISRELATEDGENES1* (*NPR1*) and *TGA* that acts together to activate the SA-induced transcription [63]. Homologs of these genes *GSMUA_Achr5T08160* (*NPR1*) and *GSMUA_Achr2T06770* (*TGA*) had higher expression in PB in the pulp of the *MaMADS2* repressed lines (Fig. 8D, F and Additional file 2: Table S2). The expression of these genes in WT pulp decreased immediately after MG and gradually during ripening in the peel. Nevertheless, the decline in expression occurs also in the pulp of the transgenic, but was delayed till B, while that in peel was similar to that in WT (Fig. 8D and Additional file 2: Table S2). The Hormonometer analysis showed a small positive correlation between the query gene pool to the transcriptome footprint of SA [45] (Fig. 9), suggesting a SA-transcriptome footprint in the transgenic lines.

## Discussion

Transcriptome analysis of peel and pulp revealed similar pattern at MG which diverged during maturation to distinct peel and pulp transcriptome, indicating that different ripening programs exist in these parts. There were almost no differences between WT and the MaMADS2 repressed lines at MG or breaker/ripe supporting that fruits from the repressed lines and WT were in a similar physiological stages at MG and B/R which indicates that repressed lines reached full ripening (Fig. 2B). The majority of differences between the MaMADS2 repressed fruit and WT appeared in the pulp of PB. In addition, the time from PB to B was extended in the repressed lines, in comparison to WT which indicates that MaMADS2 has a role in enhancing the ripening progress. This further supports our previous conclusion that MaMADS2 determines the rate of ripening [4]. Since GO analysis identified functional groups related to hormonal regulation which were significantly changed in the pulp of *MaMADS2* repressed lines in comparison to WT (Fig. 2D), it is suggested that MaMADS2 coordinate the hormone response of ethylene, ABA, auxin, cytokinin, gibberellins, jasmonic acid and salicylic acid prior to the breaker stage.

Ethylene biosynthesis was lower and delayed in the *MaMADS2* repressed lines in comparison to WT (Fig. 1), and it was in agreement with the expression of a system II tomato *SlACS2* homolog that was suppressed at PB in *MaMADS2* repressed lines, but increased later on. It is also in agreement with lower expression in the pulp of the repressed lines of few of the *ACO* genes (Fig. 3). The expression of both *ACS* and *ACO* genes was lower also in tomato *SEPALLATA SlMADS-RIN* (*rin*) mutants [64, 65]. In tomato the SlMADS-RIN binds directly to promoters of *ACS* genes and not *ACO* genes, but it is not clear if MaMADS2 acts in a similar way. It has been demonstrated that the ERF11 in banana recruits histone deacetylase to the promoters of *ACO1* gene and most likely deactivates its expression [48]. In agreement with lower ethylene production in *MaMADS2* repressed lines, this gene is up regulated in these lines.

Another gene that was modified in tomato *rin* mutants is the negative regulator *CTR1* [66]. Similar to *rin* mutant, in *MaMADS2* repressed lines the expression of *CTR1* homolog was higher in PB compared to WT. However, while the expression in banana is reduced towards breaker, in tomato it is not [67], and it is induced by ethylene [68].

ABA signaling response gene expression and hormone levels revealed a crosstalk between ABA, ethylene and MaMADS2. In the transgenic plants, low levels of ethylene and low *MaMADS2* expression [4], coexist with high levels of ABA at all ripening stages (Figs. 1 and 4). To exclude the possibility that ethylene may affect ABA levels and the low levels of ethylene which occur in the transgenic plants might cause the ABA increase, ethylene application to MG banana fruits did not alter the levels of ABA (Fig. 4E). Similarly, treatment of tomato fruits with ACC, a precursor of ethylene, did not alter ABA levels [22], indicating there is no direct effect of ethylene on ABA synthesis (Fig. 4F). However, since application of ABA reduce the time to ethylene induction and increased ethylene production, and also increased the levels of *MaMADS2* expression (Fig. 4C), we suggest that ABA acts via an increase in *MaMADS2* expression to enhance ethylene biosynthesis and ripening. This mechanism may explain results in tomato and banana showing that ABA increased ethylene production [21, 22, 24].

Higher level of ABA was observed in *MaMADS2* repressed fruit, suggests that ABA production in banana fruit is under autoinhibitory regulation via MaMADS2 (Fig. 4F). The expression of *NECD* encoding genes responsible for ABA synthesis may explain both the increase in ABA during ripening and the higher levels in repressed lines, since the expression of one gene was increased at PB in WT and another was higher in the repressed lines than in WT.

The genes within the ABA signaling pathway most likely are negatively regulated by *MaMADS2*-box gene, since the reduction in their expression is delayed in the repressed lines. Likewise, in strawberry, a non-climacteric fruit, the SnRK2 transcript level decreased during ripening [69]. Higher levels of ABA and higher expression of the genes within the signaling response pathway in the transgenic lines might explain the high ABA-transcriptomic footprint which exists in the *MaMADS2* repressed lines (Fig. 9).

Most surprising are the results of cytokinin signal transduction and levels. Cytokinins action is usually associated with delayed senescence and fruit ripening [18], but our data showed the zeatin levels are lower in the repressed line than in WT at MG (Fig. 5B), suggesting that MaMADS2 enhances cytokinins levels prior to climacteric stage. Interestingly, CKs levels were highest in the red sectors (in comparison to green sectors) of transgenic plants overexpressing *ipt* gene involved in cytokinin biosynthesis [32]. In agreement with a positive role for cytokinin in ripening, The *CRE* genes, encoding cytokinin receptors, were higher in WT than in the repressed lines (Fig. 5A and Additional file 1: Fig. S4). In addition, the reduced expression in WT of *A-ARR* during maturation and the high levels in the transgenic plants further support a positive role of cytokinin in ripening. The *A-ARR* is induced by CK and overexpression of type *A-ARR*s represses their own expression [56], leading to shutting down of the CK response [57]. This mechanism might be augmented in the repressed lines, and as can be expected in this case, cytokinin-related transcriptomic footprint is low in those fruits (Fig. 9). It has been demonstrated in Arabidopsis that a components of the CK response pathway (ARR2) play a role in signaling of both CK and ethylene [57]. Hence, it is possible that higher CK action in WT leads to higher ethylene production by induction or shutdown of proteins leading to stabilization or degradation, respectively, of ACS homologous enzymes [70, 71]. Taken together, our results strongly suggest a positive role for CK in ripening.

Although accumulating evidence suggest a role for auxin in fruit ripening [16], the exact role is still not clear. Auxin level was high in MG pulp and declined at PB. This result is in agreement with reports in tomato and grapes where an auxin peak was observed prior to ripening and decreased during later maturation [18, 21, 72]. In contrast to banana *MaMADS2* repressed lines, in the homologous tomato *rin* and apple *MdMADS8/9* repressed lines, there were higher auxin levels in the than in WT [73, 74]. These differences might results from the sampling of more mature fruit in our study or due to species specific differences.

Repressing the expression of the *MaMADS2* affected differently the two auxin signaling pathways; on one hand, it increased the expression of genes within the TIR-AUX/IAA-ARF pathway and on the other hand, it reduced the expression of genes in IAA-induced protein translation pathway. The lower expression of genes within the latest pathway in the repressed banana pulp might impair the translation of upstream open reading frames (uORF) containing mRNAs [75]. Interestingly, it has been suggested that the translation of few proteins in the ethylene response pathway are regulated by uORFs [76]. In summary, our results suggest that in the *MaMADS2* repressed lines, auxin activity lingers on at PB, although levels decrease at this stage. This is consistent with the hypothesis developed based on data in grape and tomato that higher auxin levels precede ethylene induction and only when auxin levels and response is reduced, it enables ripening to proceed [8, 13, 14].

GA and SA were shown to delay ripening and JA to promote it [8, 18, 27, 33, 77]. Our data provide molecular support to a role for MaMADS2 in mediating the ripening-related alteration in the levels of these hormone, however, their role in banana fruit ripening remains unclear.

## Conclusions

The majority of differences in WT versus *MaMADS2* repressed fruit reflected slower acquisition of ripening phenotypes that together is manifested in delayed ripening. That is, transcriptome changes in the repressed lines generally follow WT but at additional days after harvest and to an overall lesser magnitude. The hormonal response-related transcriptome and hormonal profiles of banana fruits from WT and *MaMADS2* repressed lines point to a major effect of MaMADS2 on multiple hormonal signaling pathways and suggest that MaMADS2 coordinate the response of the different hormones, besides ethylene, in the regulation of fruit ripening. Moreover, this study suggests an intricate crosstalk between ABA and MaMADS2 and a positive role for cytokinin in fruit ripening. Since the regulation of hormonal response during ripening was rarely investigated in fleshy fruit, this study open further investigations.

## Additional files

Additional file 1: **Figure S1.** Number of genes significantly change between #32 or #44 and WT pulp at PB. **Fig. S2.** Gene Ontology (GO) groups of significantly lower (A) or higher (B) at PB in *MaMADS2* repressed fruits than in WT. **Fig. S3.** Expression of *ACO* homologs in banana fruits during ripening. **Fig. S4.** Expression of *CRE1* homologs in banana fruits during ripening. **Fig. S5.** Expression of *TIR* homologs in banana fruits during ripening. **Fig. S6.** Expression of *DELLA* homologs (A) and levels of GA_9_ (B) in banana fruits during ripening. **Table S1.** Banana genes annotation and chromosome location. (PPTX 833 kb)

Additional file 2: **Table S2.** BaseMean levels of the hormone-related genes in banana fruits during ripening. U-pulp, P-peel, H-harvest (MG), PB-prebreaker, B-breaker, R-ripe. (XLSX 21 kb)

## Abbreviations

ABA: Abscisic acid
B: Breaker
CK: Cytokinin|
GA: Gibberellin
IAA: Indole acetic acid
JA: Jasmonic acid
MG: Mature Green
PB: Prebreaker
SA: Salicylic acid
