# Supplementary material for: MaMADS2 repression in banana fruits modifies hormone synthesis and signalling pathways prior to climacteric stage
Source: BMC Plant Biol. 2018 Nov 6;18:267. doi: 10.1186/s12870-018-1480-5 (PMC6219179; doi:10.1186/s12870-018-1480-5)
Supplement: Supplementary file 1 — Figure S1. Number of genes significantly change between #32 or #44 and WT pulp at PB. Fig. S2. Gene Ontology (GO) groups of significantly lower (A) or higher (B) at PB in MaMADS2 repressed fruits than in WT. Fig. S3. Expression of ACO homologs in banana fruits during ripening. Fig. S4. Expression of CRE1 homologs in banana fruits during ripening. Fig. S5. Expression of TIR homologs in banana fruits during ripening. Fig. S6. Expression of DELLA homologs (A) and levels of GA9 (B) in banana fruits during ripening. Table S1. Banana genes annotation and chromosome location. (PPTX 833 kb) [file 12870_2018_1480_MOESM1_ESM.pptx]

## Slide 1
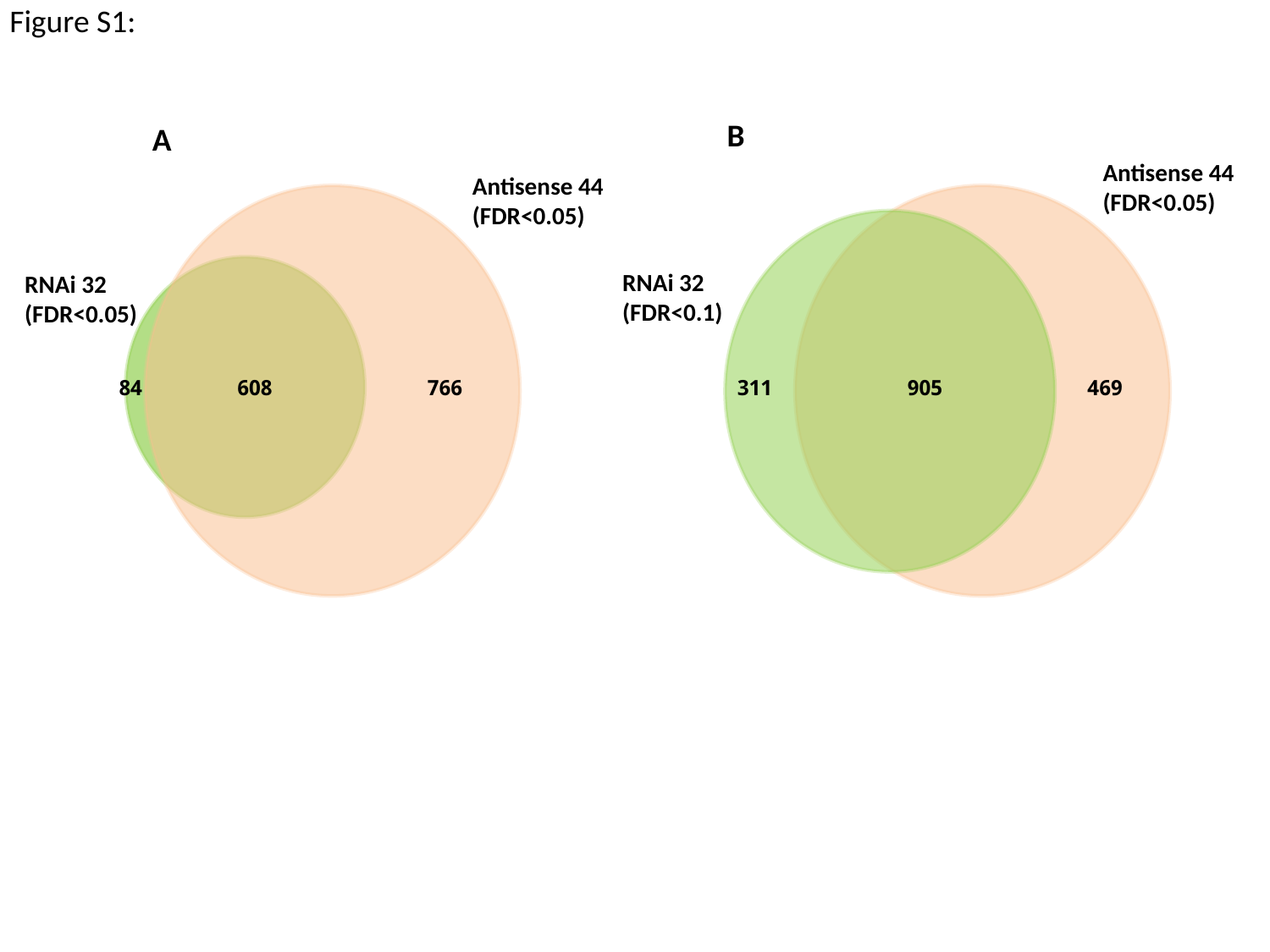

Figure S1:
B
A
Antisense 44 (FDR<0.05)
Antisense 44 (FDR<0.05)
RNAi 32 (FDR<0.1)
RNAi 32 (FDR<0.05)
84
608
766
311
905
469

## Slide 2
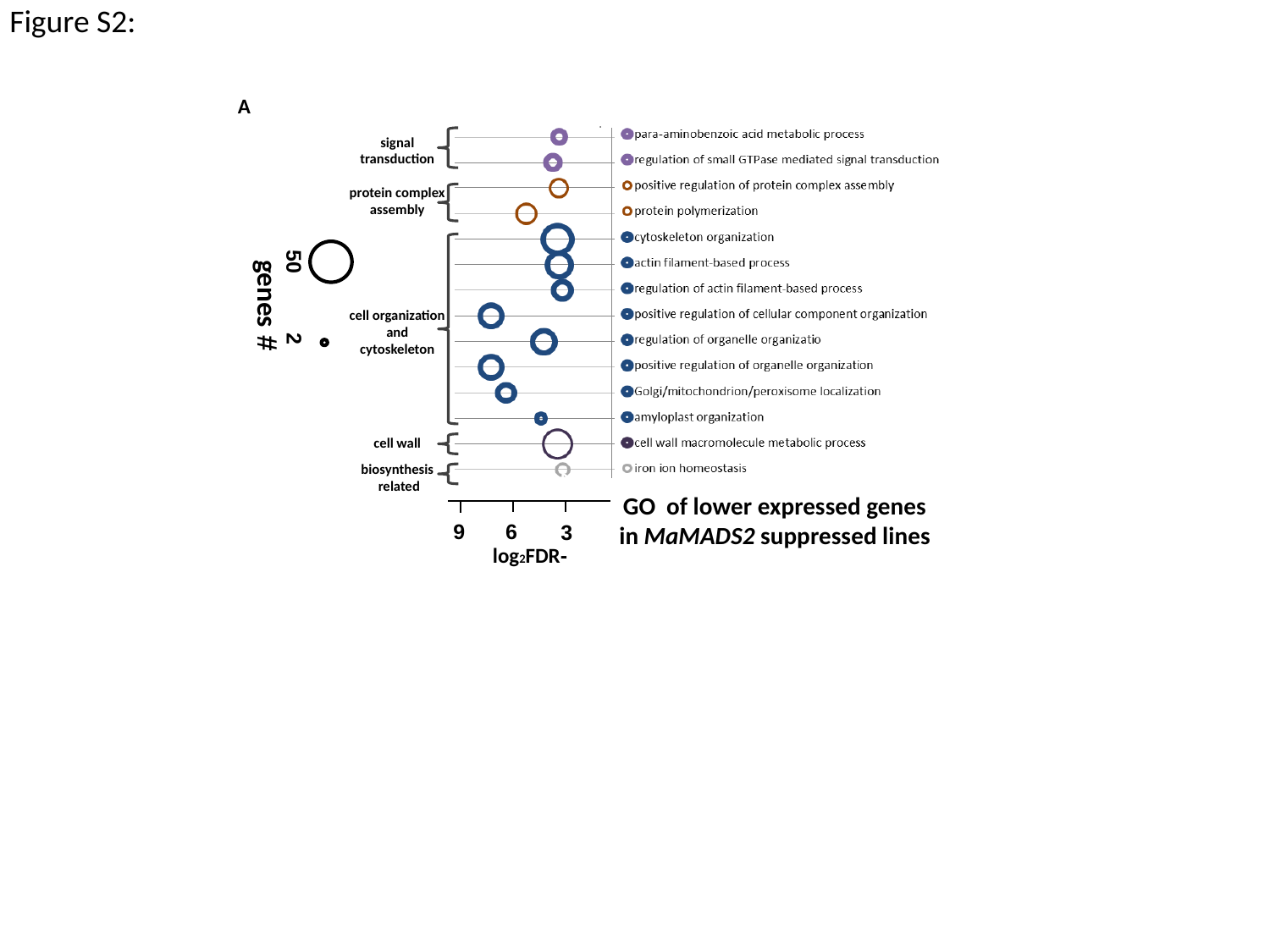

Figure S2:
A
signal transduction
protein complex assembly
50
# genes
cell organization and cytoskeleton
2
cell wall
biosynthesis related
44
GO of lower expressed genes in MaMADS2 suppressed lines
9
6
3
-log2FDR

## Slide 3
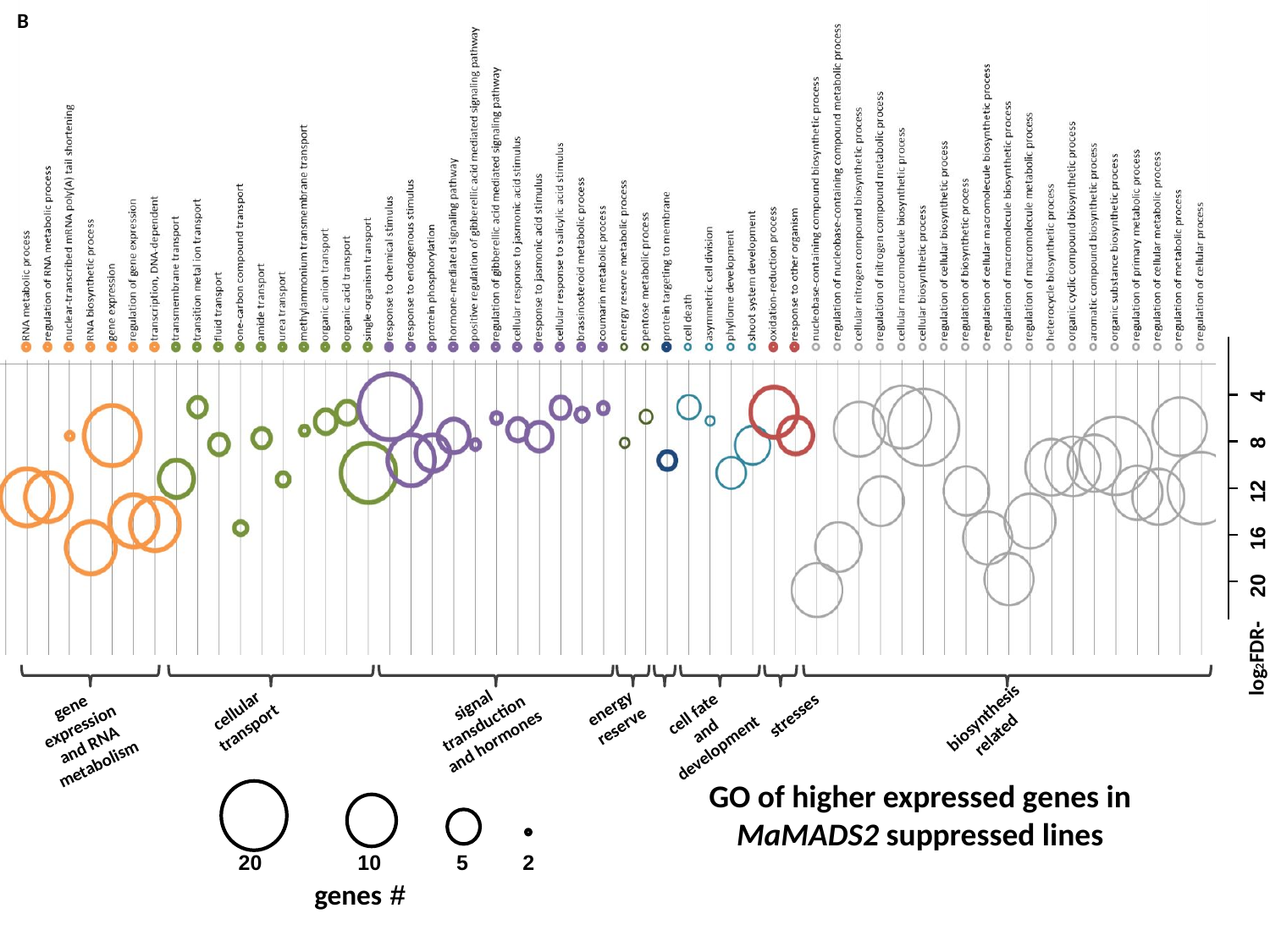

signal transduction and hormones
gene expression and RNA metabolism
energy reserve
cellular transport
cell fate
and
development
biosynthesis related
stresses
B
4
8
12
16
20
-log2FDR
GO of higher expressed genes in MaMADS2 suppressed lines
200
100
50
2
# genes

## Slide 4
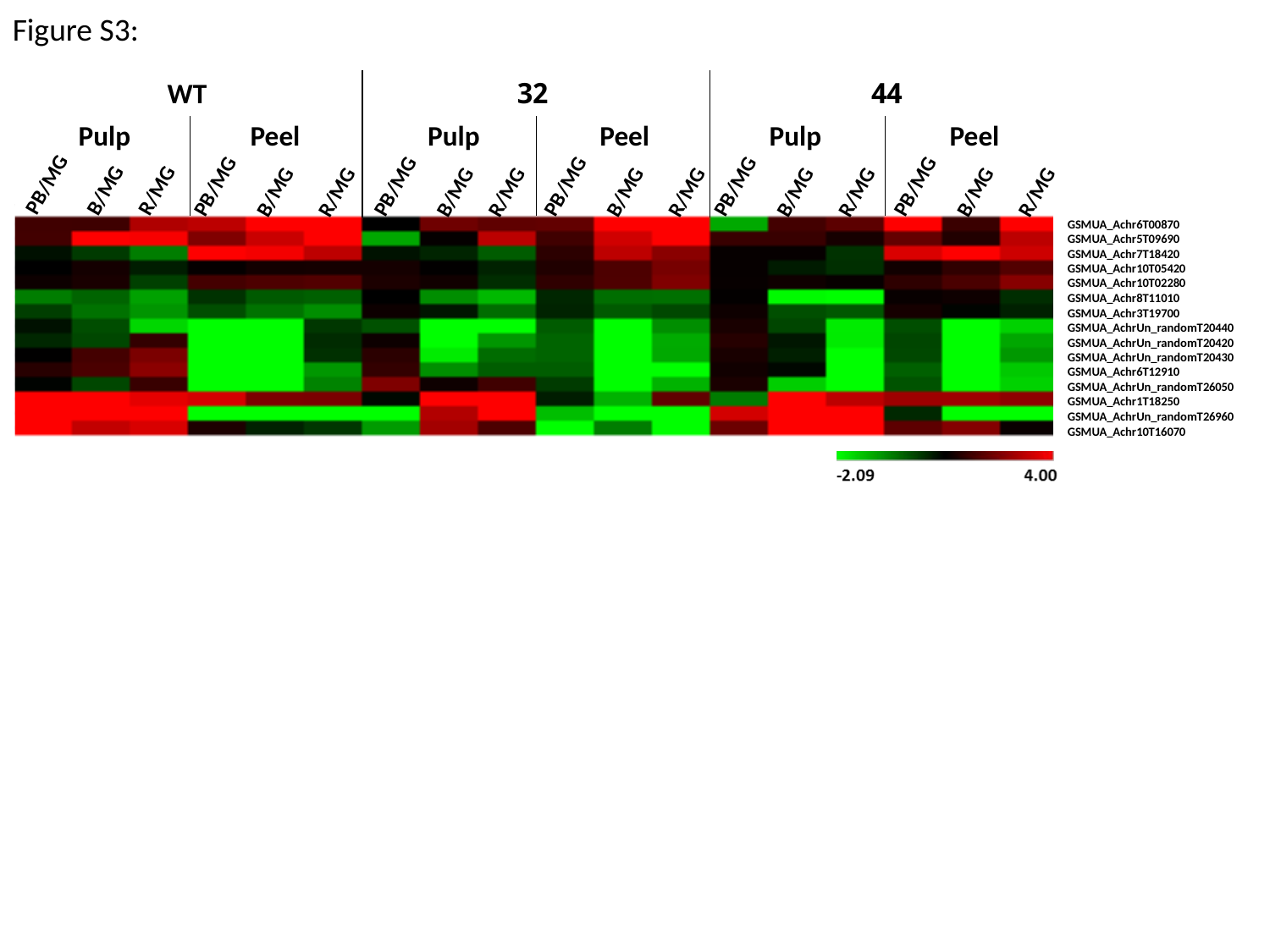

Figure S3:
WT
32
44
Pulp
Peel
Pulp
Peel
Pulp
Peel
PB/MG
PB/MG
PB/MG
PB/MG
PB/MG
PB/MG
B/MG
R/MG
B/MG
B/MG
B/MG
B/MG
B/MG
R/MG
R/MG
R/MG
R/MG
R/MG
GSMUA_Achr6T00870
GSMUA_Achr5T09690
GSMUA_Achr7T18420
GSMUA_Achr10T05420
GSMUA_Achr10T02280
GSMUA_Achr8T11010
GSMUA_Achr3T19700
GSMUA_AchrUn_randomT20440
GSMUA_AchrUn_randomT20420
GSMUA_AchrUn_randomT20430
GSMUA_Achr6T12910
GSMUA_AchrUn_randomT26050
GSMUA_Achr1T18250
GSMUA_AchrUn_randomT26960
GSMUA_Achr10T16070

## Slide 5
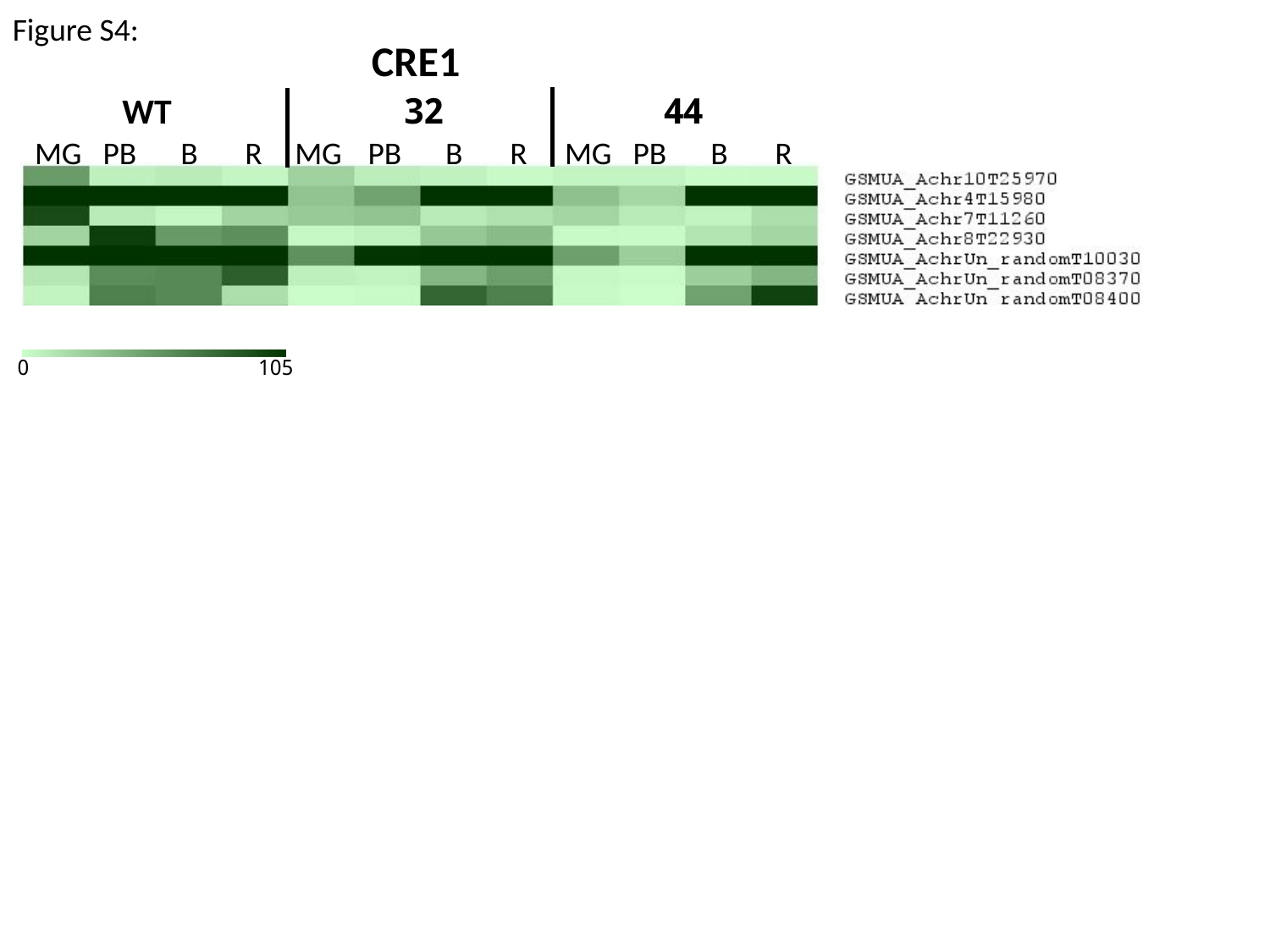

Figure S4:
CRE1
WT
32
44
MG
PB
B
R
MG
PB
B
R
MG
PB
B
R
0
105

## Slide 6
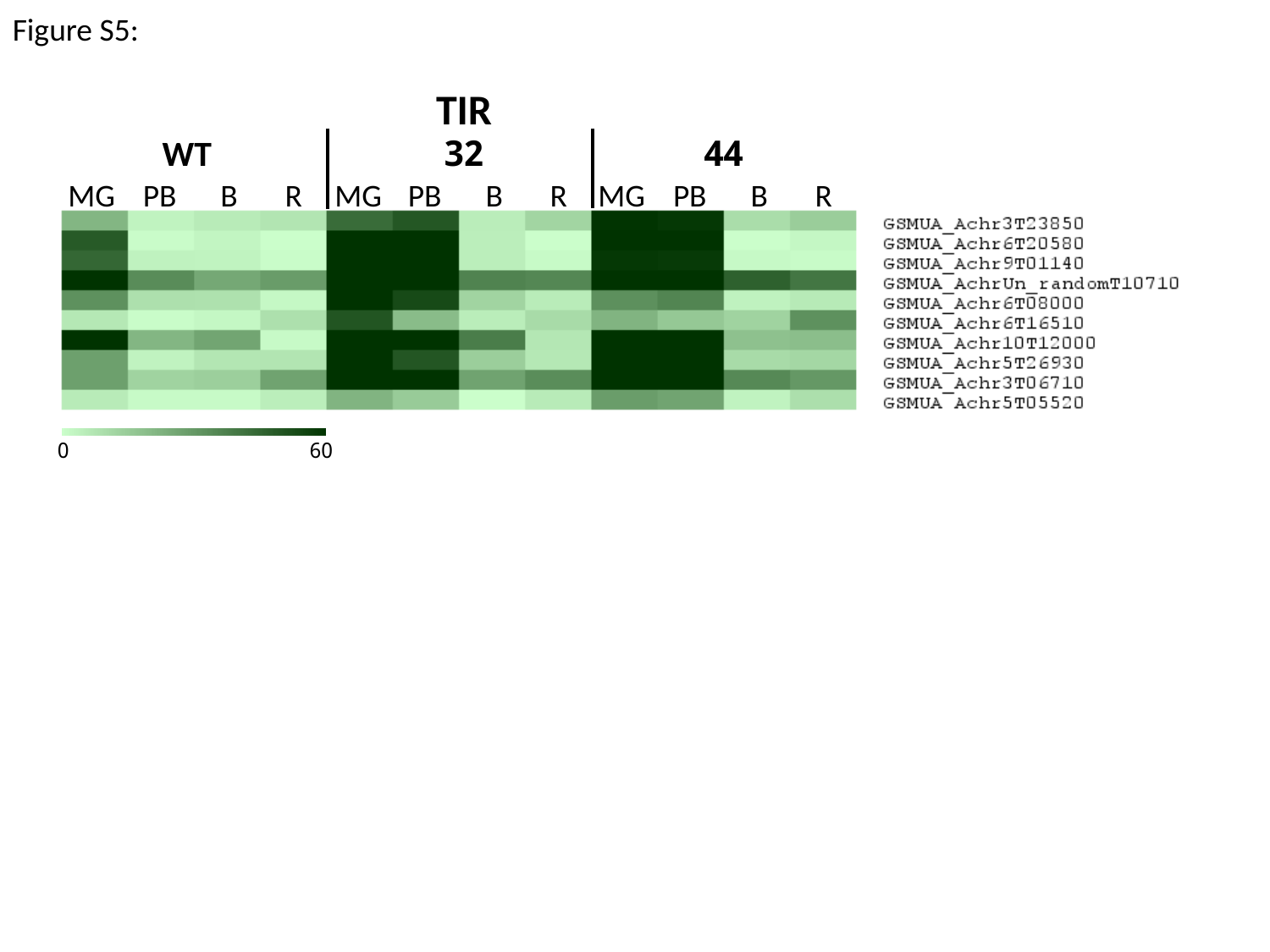

Figure S5:
TIR
WT
32
44
MG
PB
B
R
MG
PB
B
R
MG
PB
B
R
0
60

## Slide 7
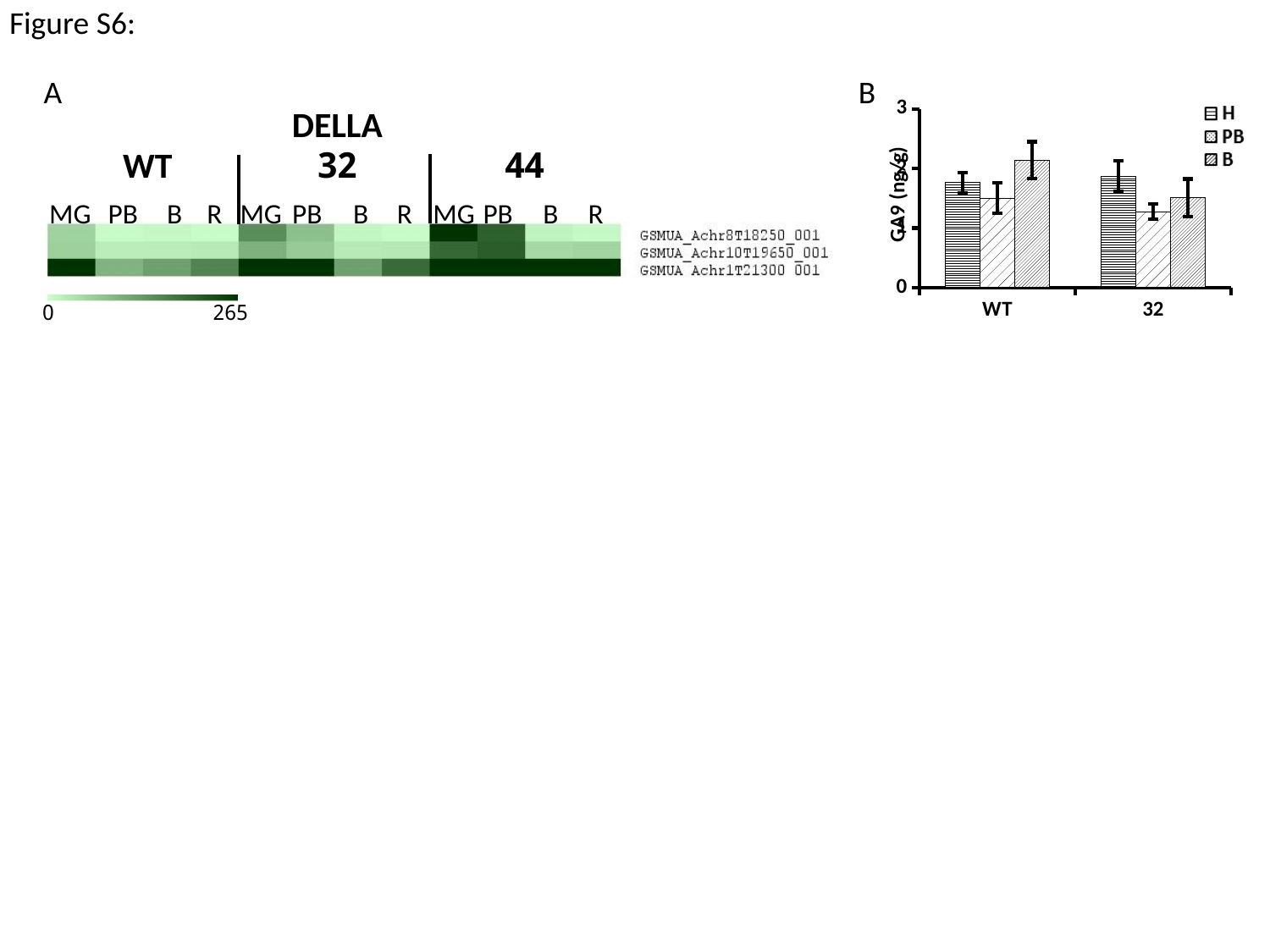

Figure S6:
A
B
### Chart
| Category | | | |
|---|---|---|---|
| WT | 1.762268480826556 | 1.501276164709396 | 2.1383748682948807 |
| 32 | 1.871651797992237 | 1.2749161897185919 | 1.5070110966616654 |DELLA
WT
32
44
MG
MG
MG
PB
B
R
PB
B
R
PB
B
R
0
265
GA9 (ng/g)

## Slide 8
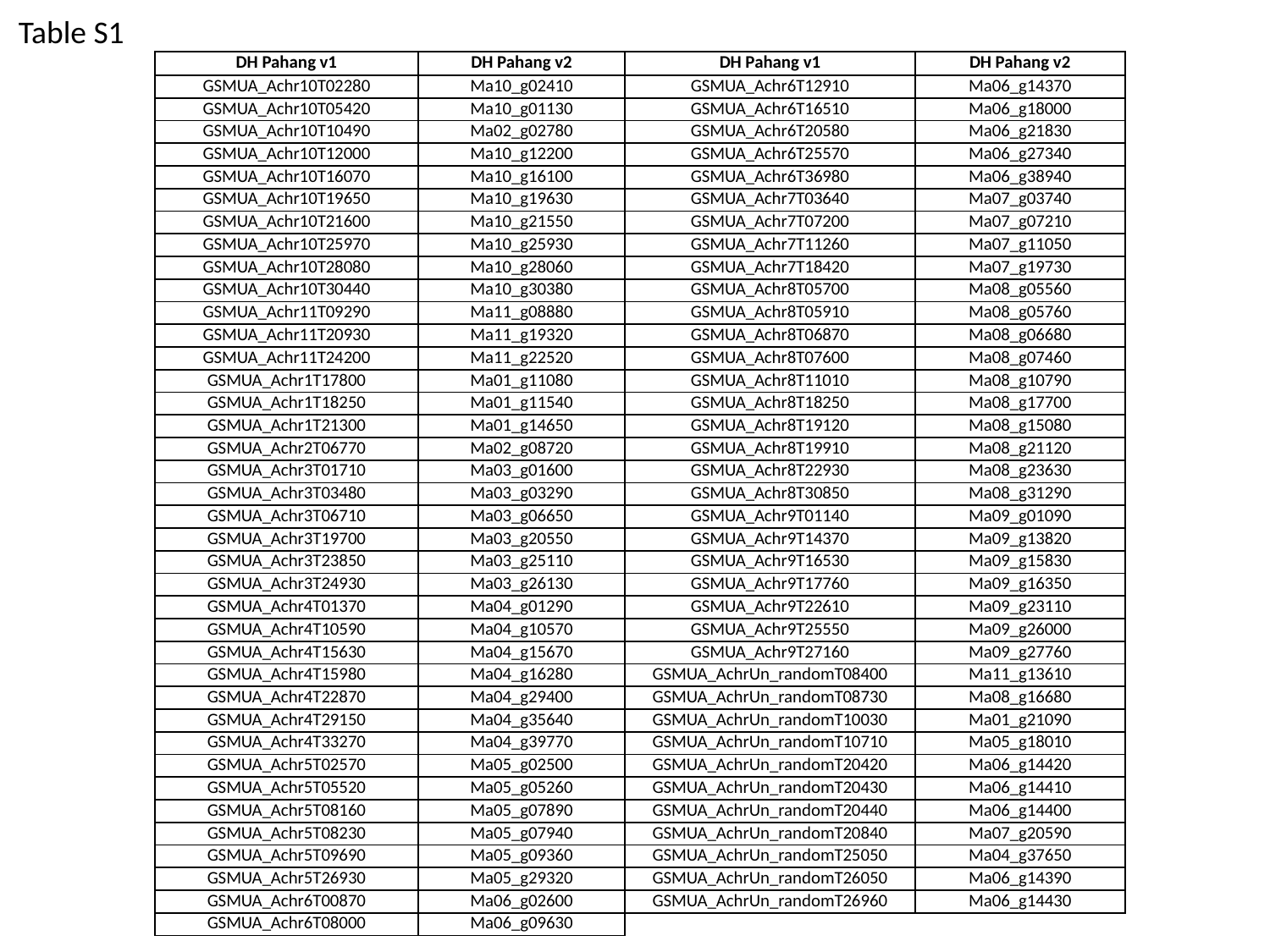

Table S1
| DH Pahang v1 | DH Pahang v2 | DH Pahang v1 | DH Pahang v2 |
| --- | --- | --- | --- |
| GSMUA\_Achr10T02280 | Ma10\_g02410 | GSMUA\_Achr6T12910 | Ma06\_g14370 |
| GSMUA\_Achr10T05420 | Ma10\_g01130 | GSMUA\_Achr6T16510 | Ma06\_g18000 |
| GSMUA\_Achr10T10490 | Ma02\_g02780 | GSMUA\_Achr6T20580 | Ma06\_g21830 |
| GSMUA\_Achr10T12000 | Ma10\_g12200 | GSMUA\_Achr6T25570 | Ma06\_g27340 |
| GSMUA\_Achr10T16070 | Ma10\_g16100 | GSMUA\_Achr6T36980 | Ma06\_g38940 |
| GSMUA\_Achr10T19650 | Ma10\_g19630 | GSMUA\_Achr7T03640 | Ma07\_g03740 |
| GSMUA\_Achr10T21600 | Ma10\_g21550 | GSMUA\_Achr7T07200 | Ma07\_g07210 |
| GSMUA\_Achr10T25970 | Ma10\_g25930 | GSMUA\_Achr7T11260 | Ma07\_g11050 |
| GSMUA\_Achr10T28080 | Ma10\_g28060 | GSMUA\_Achr7T18420 | Ma07\_g19730 |
| GSMUA\_Achr10T30440 | Ma10\_g30380 | GSMUA\_Achr8T05700 | Ma08\_g05560 |
| GSMUA\_Achr11T09290 | Ma11\_g08880 | GSMUA\_Achr8T05910 | Ma08\_g05760 |
| GSMUA\_Achr11T20930 | Ma11\_g19320 | GSMUA\_Achr8T06870 | Ma08\_g06680 |
| GSMUA\_Achr11T24200 | Ma11\_g22520 | GSMUA\_Achr8T07600 | Ma08\_g07460 |
| GSMUA\_Achr1T17800 | Ma01\_g11080 | GSMUA\_Achr8T11010 | Ma08\_g10790 |
| GSMUA\_Achr1T18250 | Ma01\_g11540 | GSMUA\_Achr8T18250 | Ma08\_g17700 |
| GSMUA\_Achr1T21300 | Ma01\_g14650 | GSMUA\_Achr8T19120 | Ma08\_g15080 |
| GSMUA\_Achr2T06770 | Ma02\_g08720 | GSMUA\_Achr8T19910 | Ma08\_g21120 |
| GSMUA\_Achr3T01710 | Ma03\_g01600 | GSMUA\_Achr8T22930 | Ma08\_g23630 |
| GSMUA\_Achr3T03480 | Ma03\_g03290 | GSMUA\_Achr8T30850 | Ma08\_g31290 |
| GSMUA\_Achr3T06710 | Ma03\_g06650 | GSMUA\_Achr9T01140 | Ma09\_g01090 |
| GSMUA\_Achr3T19700 | Ma03\_g20550 | GSMUA\_Achr9T14370 | Ma09\_g13820 |
| GSMUA\_Achr3T23850 | Ma03\_g25110 | GSMUA\_Achr9T16530 | Ma09\_g15830 |
| GSMUA\_Achr3T24930 | Ma03\_g26130 | GSMUA\_Achr9T17760 | Ma09\_g16350 |
| GSMUA\_Achr4T01370 | Ma04\_g01290 | GSMUA\_Achr9T22610 | Ma09\_g23110 |
| GSMUA\_Achr4T10590 | Ma04\_g10570 | GSMUA\_Achr9T25550 | Ma09\_g26000 |
| GSMUA\_Achr4T15630 | Ma04\_g15670 | GSMUA\_Achr9T27160 | Ma09\_g27760 |
| GSMUA\_Achr4T15980 | Ma04\_g16280 | GSMUA\_AchrUn\_randomT08400 | Ma11\_g13610 |
| GSMUA\_Achr4T22870 | Ma04\_g29400 | GSMUA\_AchrUn\_randomT08730 | Ma08\_g16680 |
| GSMUA\_Achr4T29150 | Ma04\_g35640 | GSMUA\_AchrUn\_randomT10030 | Ma01\_g21090 |
| GSMUA\_Achr4T33270 | Ma04\_g39770 | GSMUA\_AchrUn\_randomT10710 | Ma05\_g18010 |
| GSMUA\_Achr5T02570 | Ma05\_g02500 | GSMUA\_AchrUn\_randomT20420 | Ma06\_g14420 |
| GSMUA\_Achr5T05520 | Ma05\_g05260 | GSMUA\_AchrUn\_randomT20430 | Ma06\_g14410 |
| GSMUA\_Achr5T08160 | Ma05\_g07890 | GSMUA\_AchrUn\_randomT20440 | Ma06\_g14400 |
| GSMUA\_Achr5T08230 | Ma05\_g07940 | GSMUA\_AchrUn\_randomT20840 | Ma07\_g20590 |
| GSMUA\_Achr5T09690 | Ma05\_g09360 | GSMUA\_AchrUn\_randomT25050 | Ma04\_g37650 |
| GSMUA\_Achr5T26930 | Ma05\_g29320 | GSMUA\_AchrUn\_randomT26050 | Ma06\_g14390 |
| GSMUA\_Achr6T00870 | Ma06\_g02600 | GSMUA\_AchrUn\_randomT26960 | Ma06\_g14430 |
| GSMUA\_Achr6T08000 | Ma06\_g09630 | | |
